# Food for thought? The effects of the Healthy Primary School of the Future on children’s educational outcomes

**DOI:** 10.1371/journal.pone.0334638

**Published:** 2026-06-24

**Authors:** Bo H. W. van Engelen, Marla T. H. Hahnraths, Bjorn Winkens, Trudie Schils, Onno C. P. van Schayck

**Affiliations:** 1 Department of Family Medicine, Care and Public Health Research Institute (CAPHRI), Maastricht University, Maastricht, The Netherlands; 2 Department of Methodology and Statistics, Care and Public Health Research Institute (CAPHRI), Maastricht University, Maastricht, The Netherlands; 3 Department of Macro, International & Labour Economics, School of Business and Economics, Maastricht University, Maastricht, The Netherlands; Public Library of Science, UNITED KINGDOM OF GREAT BRITAIN AND NORTHERN IRELAND

## Abstract

**Background:**

There is limited empirical evidence regarding the effects of school-based health-promoting interventions on educational outcomes, highlighting a need for further research in this area to understand their broader impact on academic performance. The Healthy Primary School of the Future (HPSF) is a Dutch intervention aimed at improving children’s health by providing healthy school lunches and structured physical activity (PA) sessions. While HPSF’s positive impact on physical health has been well-documented, its effects on academic outcomes, particularly in mathematics and reading comprehension, remain less understood. This study evaluated the influence of HPSF on children’s performance by using national standardised tests for these academic domains.

**Methods:**

A longitudinal quasi-experimental design was employed involving eight Dutch primary schools over a four-year period. Schools included two full HPSF schools (implementing both PA and healthy lunches), two partial HPSF schools (PA only), and four control schools (all in the same region). Scores from biannually taken national standardised tests in mathematics and reading comprehension were combined within the same year to decrease the number of missing values. The data were analysed using mixed model for repeated measures to assess the intervention effects over time.

**Results:**

Children in full HPSF schools demonstrated significant improvements in mathematics performance compared to control schools (standardised effect size (ES) = 0.30, p = 0.011 after one year, increasing to ES = 0.66, p < 0.001 after four years). Mathematics gains in partial HPSF schools were smaller and non-significant (ES ≤ 0.23, p ≥ 0.137). For reading comprehension, small but significant improvement was observed in year 1 for full HPSF schools compared with control schools (ES = 0.28, p < 0.001), but this effect was diminished by year 4. Partial HPSF schools showed sustained small gains in reading comprehension over time (ES between 0.06 and 0.39).

**Conclusions:**

The HPSF intervention significantly enhanced mathematics performance when both healthy lunches and PA sessions were implemented, underlining the importance of a holistic approach to health promotion in schools. However, the intervention’s impact on reading comprehension was limited, indicating the need for additional and/or more targeted strategies to improve literacy outcomes. These findings highlight the potential of integrated health interventions to boost academic performance and address both health and educational inequities.

## Introduction

Several studies show that school performance of Dutch children has been declining in recent years and nationally set target levels are not met [1]. Both reading skills of primary school children aged 9–10 years and 11–12 years declined compared to earlier cohorts [1,2]. National goals are that at the end of primary school, at least 85 percent of the children reach a basic or fundamental level of reading and language skills and at least 65 percent reaches a target level for these skills. While over 90 percent of the children met the basic level between 2015 and 2022, the target level is not met in all domains of language skills [3–6]. Similar, for mathematics the majority of Dutch primary school children currently do not achieve both the basic and the target level.

Over the last years, children and adolescents are often the focus in health research [7]. As lifestyle behaviours formed during childhood are likely to track into adulthood, health promotion during childhood could not only lead to short-term health benefits for the child, but could ultimately also lead to a healthier adult population [8–10]. This idea, together with the notion that the health status of children in Western countries (including the Netherlands) is currently suboptimal across various domains (e.g., weight status, physical activity (PA), and dietary habits) [11–13] has led to the development, implementation, and evaluation of various health-promoting interventions targeting children. These interventions are often implemented in influential settings such as the school setting, where children spend a considerable amount of their time for several critical developmental years [14,15].

The effects of school-based health-promoting interventions have been investigated on a wide range of health outcomes such as weight status and dietary and PA behaviours [16–20]. However, evidence on the effects of school-based health promotion on children’s educational outcomes is limited [14], despite the strong association between children’s health and their learning capacity [21]. In the study of Schwartz and Rothbart, where free school lunches were provided, a significant improvement in academic outcomes, particularly in mathematics and reading test scores was observed [22]. Additionally, the study by Asigbee et al. (2018) revealed that higher PA levels and proper nutrition were significantly associated with improved academic performance, with students in the healthy nutrition group scoring higher on standardised tests in reading, mathematics, and science [23].

The Healthy Primary School of the Future (HPSF) is a Dutch intervention aiming to integrate health in the primary school system [24]. Between 2015–2019, the effects of HPSF were investigated in an efficacy trial involving eight Dutch primary schools: two full intervention schools, where a daily healthy school lunch was provided and structured PA sessions during the school day were incorporated, two partial intervention schools, where only the structured PA sessions were offered, and four control schools, which continued with their regular curriculum that is common practice in the Netherlands [24]. Analyses revealed significant positive intervention effects on children’s body mass index (BMI) z-score, waist circumference, and dietary and PA behaviours [25–27]. However, it remained unknown what the intervention’s effects were on children’s educational outcomes. Considering HPSF’s positive impact on children’s health and the strong link between children’s health and their learning capacity [21], it was hypothesised that children from schools implementing HPSF might have better educational outcomes than children from primary schools not implementing HPSF.

Using the results from national standardised tests [28], the present study aimed to answer the following research questions:

- What is the effect of the daily provision of a healthy school lunch combined with structured PA sessions (full HPSF), compared with PA sessions alone (partial HPSF), versus no intervention (control), on children’s performance in national standardised mathematics tests in primary schools?
- What is the effect of healthy lunch sessions combined with PA sessions (full HPSF), compared to PA sessions alone (partial HPSF), versus no intervention (control), on children’s performance in national standardised reading comprehension tests in primary schools?

## Materials and methods

### Study design

The study had a longitudinal quasi-experimental design and involved eight Dutch primary schools: two full intervention schools (full HPSFs), two partial intervention schools (partial HPSFs), and four control schools. These schools were all recruited to participate in the HPSF four-year efficacy trial based on voluntary participation [24]. The implementation of the HPSF started in November 2015 and annual measurements were performed yearly up and until 2019. The main outcome parameters were student’s scores on national standardised tests in mathematics and reading comprehension. These tests are administered to all students twice a year, normally in the middle of the school year in January/February and at the end of the school year in June.

### The healthy primary school of the future

HPSF was an intervention developed by the schoolboard of the participating schools, the regional public health services, and Maastricht University with the aim to sustainably integrate health in the school system [24]. The intervention consisted of two main changes: 1) daily provision of a free healthy school lunch, and 2) daily structured 60-minute PA sessions after lunch.

In all intervention schools, the school day was extended, which provided the opportunity to prolong the lunch break time with 45–75 min. In full HPSFs, a healthy school lunch was provided to all students, which is in sharp contrast with the normal situation in the Netherlands where students need to bring their lunch from home or go home to eat lunch. The school lunch was developed by a dietician, provided by catering services, and varied every ten weeks. At least 80% of the provided products met the dietary guidelines of the Dutch Health Council [29]. The lunch was provided in buffet style, allowing children to choose from various available food products. Children in both the full and partial HPSFs participated in structured PA sessions during lunch break time. These sessions were guided by teachers, pedagogical staff, and/or members of local sports clubs. Control schools continued with their regular curriculum that is common practice in the Netherlands.

The need for Medical Ethical approval has been waived by the Zuyderland Medical Ethics Committee in Heerlen (METCZ:14N-142). Children were allowed to participate in the measurements if their parents or guardians signed an informed consent. Data on educational performance described in this paper stems from an already existing data infrastructure that is granted with ethical approval by the Ethical Review Committee Inner City faculties. Permission to collect the data was granted (ERCIC_092_12_07_2018).

### Study population

The schools included in the current study were member of one schoolboard and were located in the Parkstad region in the southern part of the Netherlands. This region is known as a low to moderate socioeconomic area that is characterised by a relatively high prevalence of chronic diseases and a low life expectancy, as compared with the rest of the Netherlands [30,31].

All students from study years 3–8 (aged 6–12 years; internationally comparable to grades 1–6) of the eight schools were included in the present study, as the Dutch standardised test system for all core parts of the curriculum (e.g., mathematics and reading comprehension) starts from study year 3.

### Data collection procedures

In the Netherlands, all primary school students participate in national standardised tests. The tests are designed by CITO, a Dutch institute for test development (Central Institute for Test Development) and are advised to be administered twice a year (mid-school year (M); January/February and end-of-school year (E); May/June) to track children’s performance on a wide range of educational domains, including reading comprehension and mathematics [1]. Teachers grade the tests using a standardised grading scheme which allows comparison of a student’s results throughout their primary school time.

Students’ test scores were retrieved through a regional monitor that is part of an ongoing research-practice-partnership between Maastricht University and primary and secondary schools in the province of Limburg: de Onderwijsmonitor Limburg. According to the passive consent principle, parents can withdraw their child anytime.

### Measures

The main outcome variables of the present study are students’ standardised test scores on the domains mathematics and reading comprehension, as these domains are likely most prone to be influenced by external factors such as the school environment and are a good representation of general knowledge (Inspectie van het Onderwijs, 2024). In the current study, the mid-school year (M) and end-of-school year (E) standardised test scores of each student were combined by calculating an average score from the M and E tests of the same year, for each time point. If one of these test results was missing, we used the remaining available test score from that time point. However, if both test results were unavailable, the data for that time point were marked as missing. During study duration, two test versions (version A and version B), which do not differ on content but on outcome scale, as well as two entry methods of the tests (digital or on paper) were used, which we accounted for in the data analyses.

### Data analysis

Analyses were performed using IBM SPSS Statistics for Windows (version 29.0.2.0, Armonk, NY: IBM Corp). Figures were created using the forestplot package within Rstudio (version 2023.12.1 + 402) and R (version 4.3.3). Categorical variables were presented by number and percentage, whereas means and standard deviations (SD) were used for numerical variables. Proportions were compared using Pearson’s chi-square tests, and mean values were compared using one-way ANOVA.If the homoscedasticity assumption (assessed using Levene’s test) was violated, Welch’s test was used for overall comparisons, and the Games-Howell test was used for pairwise comparisons.

For the scores on mathematics and reading comprehension, the number of students for whom data were available and the observed mean and standard deviation (SD) per intervention group (full HPSF, partial HPSF, control) after one, two, three, and four years of exposure to HPSF were computed. To assess the intervention effects at different timepoints, a mixed model for repeated measures with intervention group (full HPSF, partial HPSF, control), exposure (1, 2, 3, 4 years), and their interaction as well as test version (version A or B), age (in years), and sex (male/female) as independent variables was used, where an unstructured covariance structure for repeated measurements was used. For mathematics, the standardised test score measured at baseline (average of the two years preceding the start of HPSF) was added to the model to correct for baseline differences. For reading comprehension, this baseline score was missing for almost all students and therefore not included in the model. The entry method of the tests (digital or on paper) was not included as a fixed factor, since there were only a few tests made on paper for mathematics, while for reading comprehension, version A was mainly digital and version B was mainly made on paper. The mixed model for repeated measures was used because it included all available data and assumed missing values to be at random (MAR), using a restricted maximum likelihood-based approach for estimation. The normality assumption of the residuals was checked per group and per timepoint (exposure) using histograms and qq-plots. For the corrected mean differences (i.e., age, sex, test version, and baseline score for mathematics). after one, two, three, and four years of exposure (delta1-delta4) with their corresponding 95% confidence intervals (CI) are presented. Standardised effect sizes (ES) were computed as the corrected mean difference at each timepoint (year of exposure) divided by the square root of the residual variance at the corresponding year of exposure, where ES = 0.2 is considered a small effect, 0.5 a medium effect, and 0.8 a large effect [32] (Cohen, 1988). Two-sided p-values ≤ 0.05 were considered statistically significant. To adjust for multiple group comparisons, the Bonferroni method was applied (significance level α = 0.05/3 = 0.0167). The syntax used for the analyses is provided in S1 Text.

## Results

In total, 4191 mathematics tests and 3857 reading comprehension tests were included in the current study. Participant flow across exposure waves is shown in Supplementary S2 Table. For mathematics, the majority of tests was conducted using test version B (full HPSF 1199, 90.3%; partial HPSF 537, 65.4%; and control 1629, 79.8%). The same applied to the reading comprehension tests (full HPSF 3094, 80.2%; partial HPSF 1189, 90.6%; and control 1436, 80%). Furthermore, a very small proportion of the mathematics tests was conducted on paper (full HPSF 2, 0.4%; partial HPSF 9, 0.2%; and control 7, 0.3%). For reading comprehension, most tests were conducted on paper (full HPSF 1191, 90.7%; partial HPSF 469, 62.7%; and control 1439, 80.1%) (Table 1).

**Table 1:** Test characteristics.

	Total		Full HPSF		Partial HPSF		Control	
	No. tests	No. tests (%)	No. tests	No. tests (%)	No. tests	No. tests (%)	No. tests	No. tests (%)
**Mathematics**								
Test version	4191		1328		821		2042	
*A*		826 (19.7)		129 (9.7)		284 (34.6)		413 (20.2)
*B*		3365 (80.3)		1199 (90.3)		537 (65.4)		1629 (79.8)
Entry method	4191		1328		821		2042	
*Paper*		18 (0.4)		2 (0.2)		9 (1.1)		7 (0.3)
*Digital*		4173 (99.6)		1326 (99.8)		812 (98.9)		2035 (99.7)
**Reading comprehension**								
Test version	3857		1313		748		1796	
*A*		763 (19.8)		124 (9.4)		279 (37.3)		360 (20.0)
*B*		3094 (80.2)		1189 (90.6)		469 (62.7)		1436 (80.0)
Entry method	3857		1313		748		1796	
*Paper*		3099 (80.3)		1191 (90.7)		469 (62.7)		1439 (80.1)
*Digital*		758 (19.7)		122 (9.3)		279 (37.3)		357 (19.9)

Abbreviations; No. = number, HPSF = Healthy Primary School of the Future.

As for sex and age of participants, the proportion of female children was close to 50% in each group (50.7% in full HPSF, 44.6% in partial HPSF, 51.3% in control; Pearson chi-square test p = 0.127), while there was a significant difference in age among the three intervention groups (Welch test p = 0.006). Pairwise comparisons showed that the children in the partial HPSF group were significantly younger (n = 307, mean age = 6.9 years, SD = 0.8) than those in the full HPSF group (n = 509, mean = 7.1, SD = 1.0, Games-Howell test p = 0.015) and in control schools (n = 786, mean = 7.1, SD = 0.9, Games-Howell test p = 0.009).

### Intervention Effects

**Fig 1:**
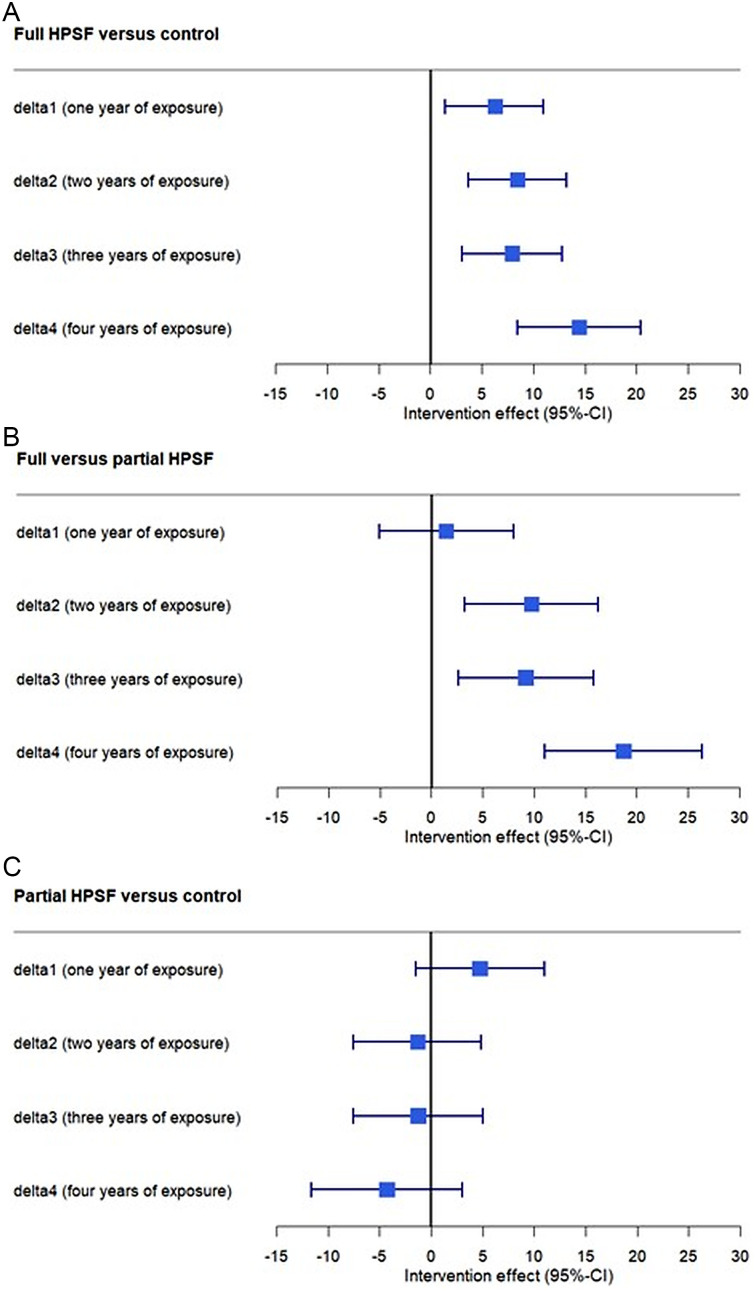
*Mathematics* – estimated intervention effect, i.e., difference in estimated means after one, two, three, and four years of exposure (delta1-delta4) corrected for outcome measured at baseline, test version, age, and sex. a) Full HPSF vs. control, b) Full HPSF vs. Partial HPSF, c) Partial HPSF vs. control. Abbreviations: HPSF = Healthy Primary School of the Future, CI = confidence interval.

All intervention effects were assessed using MMRM analysis, where all assumptions were met. The overall interaction between group and exposure was statistically significant for both mathematics and reading comprehension (see Supplementary S1 Table for full model results). The full HPSF group showed a significant improvement in mean mathematics score compared to the control group in the first year (corrected mean difference = 6.2, 95%CI 1.4 to 10.9, p = 0.011), corresponding to a small-to-medium ES of 0.30. This effect remained significant (all p ≤ 0.001) for the following years, with ES ranging from 0.37 to 0.66 (Table 2 and Fig 1a).

**Table 2:** Estimated intervention effects (corrected mean differences (B), 95% confidence intervals, two-sided p-values, and ES) after one, two, three and four years of exposure (delta1-delta4) with correction for baseline (mathematics) and age, sex, and test version (mathematics and reading comprehension).

		Full HPSF vs. control			Full HPSF vs. partial HPSF			Partial HPSF vs. control		
		B (95% CI)	*p*	ES	B (95% CI)	*p*	ES	B (95% CI)	*p*	ES
**Mathematics**	**delta1**	6.182 (1.418, 10.945)	**0.011**	0.30	1.449 (−5.098, 7.996)	0.664	0.07	4.732 (−1.512, 10.977)	0.137	0.23
	**delta2**	8.434 (3.689, 13.178)	**<0.001**	0.40	9.756 (3.233, 16.279)	**0.003**	0.47	−1.323 (−7.532, 4.887)	0.676	0.06
	**delta3**	7.948 (3.104, 12.792)	**0.001**	0.37	9.208 (2.628, 15.789)	**0.006**	0.43	−1.260 (−7.552, 5.032)	0.694	0.06
	**delta4**	14.425 (8.448, 20.403)	**<0.001**	0.66	18.713 (11.057, 26.369)	**<0.001**	0.86	−4.287 (−11.646, 3.072)	0.253	0.20
**Reading Comprehension**	**delta1**	2.690 (1.176, 4.206)	**<0.001**	0.28	−0.012 (−1.933, 1.910)	0.991	0.001	2.701 (0.895, 4.508)	**0.003**	0.28
	**delta2**	0.934 (−0.300,2.169)	0.138	0.09	−1.401 (−2.986, 0.183)	0.083	0.14	2.336 (0.800, 3.872)	**0.003**	0.24
	**delta3**	0.670 (−0.691, 2.031)	0.334	0.06	−0.037 (−1.822, 1.748)	0.968	0.003	0.707 (−0.965, 2.379)	0.407	0.06
	**delta4**	1.511 (−0.246, 3.267)	0.092	0.12	−3.507 (−5.780, −1.233)	**0.003**	0.27	5.017 (2.893, 7.141)	**<0.001**	0.39

Abbreviations; HPSF = Healthy Primary School of the Future, CI = confidence interval, ES = effect size.

The full HPSF group also showed a significant improvement in mathematics score compared to the partial HPSF group in years 2–4 (all p ≤ 0.006) with medium to large ES (0.47, 0.43, and 0.86 for years 2, 3, and 4, respectively) (Table 2 and Fig 1b). The partial group showed no significant improvement in mathematics score compared to the control group (Table 2 and Fig 1c).

**Fig 2:**
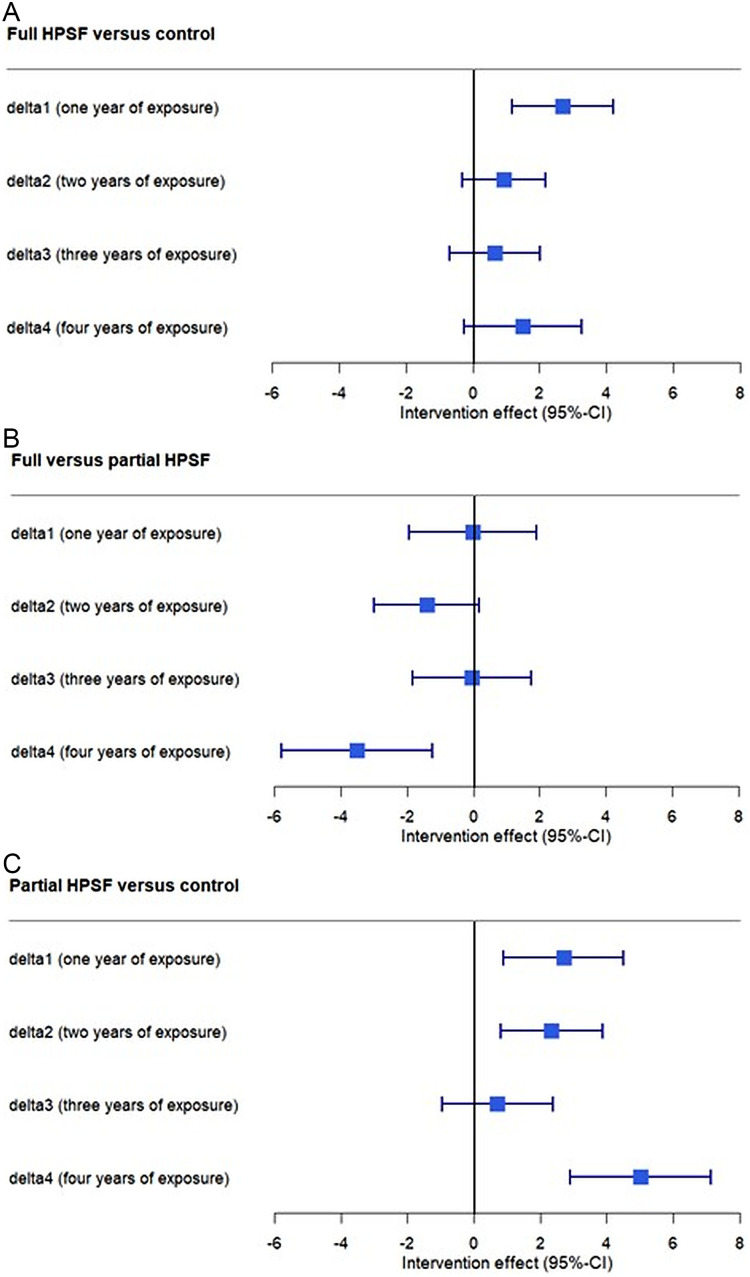
*Reading Comprehension* – estimated intervention effect, i.e., difference in estimated means after one, two, three, and four years of exposure (delta1-delta4) corrected for test version, age, and sex. a) Full HPSF vs. control, b) Full HPSF vs. Partial HPSF, c) Partial HPSF vs. control. Abbreviations: HPSF = Healthy Primary School of the Future, CI = confidence interval.

As for reading comprehension, the full HPSF group showed only a significant intervention effect compared to the control group in year 1 (corrected mean difference = 2.7, 95%CI 1.2 to 4.2, p < 0.001), which corresponded to an ES of 0.28 (Table 2 and Fig 2a). The full HPSF group had a significantly lower mean reading comprehension score than the partial HPSF group after 4 years (corrected mean difference = −3.5, 95%CI −5.8, −1.2, p = 0.003) with a small-to-medium ES of 0.27 (Table 2 and Fig 2b). In addition, the partial HPSF group scored significantly higher than the control group in year 1, 2, and 4 (all p ≤ 0.003) with small-to-medium ES of 0.28, 0.24, and 0.39, respectively (Table 2 and Fig 2c).

For the observed mean test scores for mathematics and reading comprehension and standard deviations for each intervention group over time, see S1 Fig 1.

## Discussion

The present study explored the impact of the HPSF intervention on children’s educational outcomes, specifically focusing on mathematics and reading comprehension performance.

### Mathematics performance

The outcomes in this study indicate that the full HPSF intervention led to significantly improved mathematics performance compared to control schools. After one year of exposure, the full HPSF group outperformed the control group (ES = 0.30, p = 0.011), and this improvement persisted and even grew stronger over time, with the highest ES observed after four years of exposure (ES = 0.66, p < 0.001). There was no significant effect of the partial HPSF group versus the control group, underpinning the importance of the combination of a healthy school lunch and structured PA sessions.

To put the effect of full HPSF versus control on mathematics score in perspective, we used the observed data from a child on a control school to compute the annual increase over time and translate this to a theoretical annual increase in case this child was on a full HPSF and additionally compare this to the national average annual increase in the same period (S2 Fig 2). From this theoretical example we can see that the child in the control group demonstrated a modest average increase of 12.9 points per year, reflecting individual progress augmented by national trends. In contrast, based on the estimated full intervention effects, this child would have a higher average annual increase of 16.5 points per year if this child went to a HPSF. Interestingly, the national average increase across the same period was 15.5 points per year, indicating that while the full HPSF group outperformed the national trend with approximately 6%, the control group fell short of it with approximately 17% (S2 Fig 2I).

Research has consistently shown that PA can enhance cognitive functions such as executive function and memory, which are critical for learning tasks, including mathematics [32, 33]. Results of a meta-analysis by Muntaner-Mas et al. showed that PA was associated with significant improvements in mathematics test scores [34].

Furthermore, previous research showed that children with healthier dietary and PA behaviours performed better on standardized tests, including mathematics, while those with unhealthy diets and low activity levels scored lower in reading, mathematics, and science, even when controlling for socioeconomic status (SES), age, and sex [23]. HPSF’s positive impact on children’s dietary behaviours has already been demonstrated [25]. Potentially, this change in dietary behaviours (in combination with increased PA) consequently positively impacted children’s concentration and cognitive functioning during academic tasks [35,36].

### Reading comprehension

In contrast to the clear improvements observed for mathematics performance, the effect of HPSF on reading comprehension was modest. After one year of exposure, a small but significant improvement was observed in the full HPSF group compared to the control group (ES = 0.28, p < 0.001), but the effect did not sustain over time. By the fourth year, the difference between the full HPSF group and the control group was no longer statistically significant (ES = 0.12, p = 0.092). There was a significant difference in mean reading comprehension scores between the partial HPSF group and the control group after one, two, and four years of exposure. However, the ES (0.06–0.39) were only ranging from very small to small-to-medium [37] and the significant difference was absent at year 3. As for the larger effect of partial HPSF over full HPSF, this difference was only statistically significant at year 4 and showed no consistent pattern (almost no difference at year 1 and year 3). This difference over time might also be partially explained by a possible difference between the partial and full HPSF schools at baseline, which could not be accounted for due to the number of missing values. This is also mentioned as one of the main limitations below.

One potential explanation for the limited effect of HPSF on reading comprehension is that this domain may be less sensitive to changes in physical health and/or nutritional status compared to mathematics performance and more depending on a broad range of external factors whilst mathematics development is more depending on the school context. For language outcomes, data from three studies demonstrated a small, positive effect of PA notably in reading, although comprehension tasks showed no significant gains [34]. Previous research has suggested that while PA and diet can positively influence general cognitive functions, specific academic domains may be influenced by additional factors, such as language exposure and literacy-related activities outside of school [32,38]. Maybe home environment (even preceding to school environment) has already led to literacy development, leaving less room for direct improvement than for mathematical development [39]. It is also possible that the benefits of health promotion on reading skills take longer to manifest, or that reading comprehension improvements require more targeted educational interventions that were not addressed by HPSF.

### Strengths and limitations

A major strength of this study is its longitudinal quasi-experimental design, which allowed for the evaluation of HPSF’s impact over a four-year period. The use of national standardised test scores, administered biannually, provided robust and reliable measures of educational outcomes. Additionally, the inclusion of both full and partial HPSF schools enabled a deeper understanding of the contribution of the combination of healthy school lunches and PA sessions to educational outcomes, as well as the specific contribution of PA sessions in isolation.

Implementation of the core components of the HPSF, including the healthy lunch and structured physical activity, was monitored through direct and intensive involvement of the research team, with regular contact at both the school and school board levels to assess whether implementation aligned with the agreed implementation plan. This multi-level approach was intended to address a well-known challenge in educational research, namely that interventions are frequently not implemented as intended. Engagement at both the school and governance levels supported internal ownership of the intervention and facilitated sustained implementation over time. Moreover, consistent with the conceptualization of the HPSF as a complex adaptive system, the accompanying process evaluation emphasized not only fidelity but also context-specific adaptations, as strict fidelity does not necessarily translate into greater effectiveness in complex educational settings

However, the study also had limitations. First, as previously indicated, the schools that participated in the current study were located in a low- to moderate socioeconomic region, which may limit the generalisability of the findings to other contexts. Second, as the present study did not include an intervention group only receiving a healthy school lunch, we cannot report on the impact of a healthy school lunch in isolation. This limits our ability to draw firm conclusions in this regard. Third, the study did not account for potential confounding factors such as SES, parental involvement, home learning environment, or extracurricular activities, all of which may influence academic performance [40].

The reason why we were unable to adjust for children’s socioeconomic background was that information on parental education (as a proxy for SES) is not routinely collected in Dutch primary education and was therefore only available via parental questionnaires. Unfortunately, a substantial proportion of parents (~50%) did not complete these questionnaires. Non-response is known to be more common among families with a lower SES or a non-Dutch language background [30]. While residual confounding cannot be excluded, several considerations may reduce this concern: (1) all schools were located in the same low-to-moderate SES region under one school board, limiting SES heterogeneity; and (2) a previous study (specifically designed to investigate the external validity of the study with the present study population [30]) confirmed that participants were broadly representative of the region in terms of sex (48% boys vs. 50% regionally) and socioeconomic status, as indicated by maternal educational level (25% low, 45% middle, 30% high vs. 27%, 42%, 31% regionally), as well as health-related characteristics. Nevertheless, replication of this study in other socioeconomic contexts is necessary to establish the generalizability of our findings.

Interpretation of effect sizes in educational outcomes was challenging. We therefore presented all results also in standardized form (Cohen’s d) to facilitate comparison. To further contextualize the findings, we illustrated the effects using a theoretical example based on observed data: Children in control schools gained on average 12.9 mathematics points per year, compared to 16.5 points in full HPSF schools per year. while the national average was 15.5 points per year. Thus, children in HPSF schools progressed faster than the national trend, whereas those in control schools fell behind (S3 Fig 3). Although the standardized effect sizes were small-to-moderate in statistical terms, such differences may accumulate over years to have meaningful educational consequences. Direct comparison with other health-based interventions is unfortunately not possible, as to our knowledge no comparable studies with standardized test outcomes have been published. Furthermore, the outcomes for reading comprehension were not corrected for the outcome measured at baseline (due to too many missing values) so we cannot draw an unequivocal conclusion. An important limitation is that key potential confounders, such as socioeconomic status, the home learning environment and reading comprehension at baseline, could not be included due to substantial missing data. As a result, residual confounding and selection bias cannot be ruled out. Schools opting into the HPSF may differ systematically from control schools in unobserved characteristics related to both health promotion and academic outcomes, which limits causal interpretation of the findings. Previous research on the HPSF cohort indicates that although evidence for a healthy-volunteer effect is limited, responders were more likely to have higher educated parents and differed from the national population on several sociodemographic characteristics. Consequently, external validity appears to be high with respect to the regional population but more limited when comparisons are made at the national level. (30) Lastly, while the study focused on mathematics performance and reading comprehension, other important domains such as language skills and social-emotional development were not assessed.

### Implications for education, health promotion, and future research

The results of this study can have important implications for educational policy, health promotion in schools, and future research. First, they highlight the potential of integrating health-promoting activities, such as providing healthy school lunches and encouraging PA, to not only improve children’s health but also to enhance academic outcomes, particularly with regard to their mathematics performance. These findings support the idea that a holistic approach to health and education can benefit multiple aspects of a child’s development [41]. As far as we know this is the first controlled study performed in this specific area and therefore, we advise replication of the study in other SES environments, as well in other countries, before more general conclusions may be drawn.

The study also revealed that the benefits of HPSF are not uniform across all academic domains, with stronger effects observed in mathematics compared to reading comprehension, as the home-learning environment was significantly associated with fluid intelligence and early language outcomes, but not with numeracy, where school-related factors played a greater role [42]. This suggests that future interventions could benefit from tailoring to target specific educational outcomes more effectively.

While some may view school health programs primarily as quality improvement, we emphasize that the HPSF was evaluated in a longitudinal quasi-experimental design with repeated, standardized national testing over four years. This methodological rigor, combined with a large dataset and robust analyses, allows us to provide scientific evidence on the academic effects of a complex school-based health intervention.

Equity in education at primary school is an important issue in education, where it is aimed that all students are given equal opportunities to discover and develop their talents, regardless of their SES, ethnicity, or other circumstances. However, differences in learning achievements and educational opportunities between children from different backgrounds are visible in the Netherlands. Factors such as home situation, school quality, and early selection in education play a role [30]. The results of this research can contribute to equality of opportunity for all primary school students, as providing a healthy school lunch and more PA may improve learning and scholastic behaviour.

Further research exploring how (healthy) nutrition and PA influence academic outcomes across different settings—such as informal education and home learning environments—could contribute to a broader understanding of the mechanisms at work. Furthermore, more research focusing solely on nutritional interventions would allow to untangle the effects of dietary factors from other variables, offering a clearer understanding of how nutrition alone can impact cognitive functioning and academic performance.

## Conclusion

In conclusion, the HPSF intervention demonstrated a strong positive influence on children’s mathematics performance when the full HPSF intervention (i.e., both healthy lunches and structured PA sessions) was implemented. The intervention effects on reading comprehension were only present for the partial HPSF group, but these were relatively small and indecisive. The overall findings support the integration of health-promoting interventions in primary schools. Through these interventions, both physical and academic development can be enhanced, which in turn can promote health and educational equity.

## Supporting information

S1 Fig Observed mean test scores (mathematics and reading comprehension) and standard deviations (SDs) for each intervention group (Full HPSF, Partial HPSF, Control) after one, two, three, and four years of exposure (Δ1, Δ2, Δ3Δ4). Abbreviations: HPSF = Healthy Primary School of the Future; SD = standard deviation.
(DOCX)

S2 Fig Theoretical example illustrating the effect of full HPSF and control on mathematics outcomes. Changes in scores per year are compared with national averages derived from the Pupil Administration System (LAS) ParnasSys database [43].
(DOCX)

S3 Fig Change in mathematics performance after one year of intervention compared with the national Dutch average. (DOCX)

S1 Text Syntax for mathematics and Dutch language analyses. Syntax file used for the analyses of mathematics and Dutch language outcomes.
(DOCX)

S1 Table Full model results. Supplementary table presenting the full mixed model results for the intervention effects on mathematics and reading comprehension.
(DOCX)

S2 Table Participant flow across exposure waves. Supplementary table presenting participant flow and available test-score data across.
(DOCX)
