# Supplementary material for: Food for thought? The effects of the Healthy Primary School of the Future on children’s educational outcomes
Source: PLoS One. 2026 Jun 24;21(6):e0334638. doi: 10.1371/journal.pone.0334638 (PMC13293421; doi:10.1371/journal.pone.0334638)
Supplement: S1 Fig — Abbreviations: HPSF = Healthy Primary School of the Future; SD = standard deviation. (DOCX) [file pone.0334638.s001.docx]

**S1 Figure 1. Observed mean test score (mathematics and reading comprehension) and SDs for each intervention group after one, two, three, and four years of exposure (delta1-delta4).**

|  | | **Full HPSF** | | **Partial HPSF** | | **Control** | |
| --- | --- | --- | --- | --- | --- | --- | --- |
|  |  | **n** | **Mean (SD)** | **n** | **Mean (SD)** | **n** | **Mean (SD)** |
| **Mathematics** | **delta1** | 212 | 147.2 (50.7) | 139 | 103.2 (48.2) | 342 | 129.2 (54.5) |
|  | **delta2** | 307 | 164.2 (52.3) | 189 | 130.7 (50.8) | 471 | 147.6 (55.2) |
|  | **delta3** | 481 | 170.4 (54.2) | 285 | 147.5 (49.5) | 733 | 158.0 (53.3) |
|  | **delta4** | 328 | 209.9 (42.1) | 208 | 182.6 (54.1) | 496 | 195.0 (47.7) |
| **Reading Comprehension** | **delta1** | 213 | 30.2 (9.5) | 135 | 25.7 (12.7) | 305 | 28.1 (11.4) |
|  | **delta2** | 449 | 31.7 (10.3) | 233 | 30.3 (9.9) | 482 | 32.5 (11.8) |
|  | **delta3** | 404 | 36.6 (12.9) | 237 | 33.8 (11.1) | 630 | 35.3 (13.0) |
|  | **delta4** | 255 | 45.4 (12.3) | 154 | 44.0 (12.5) | 392 | 41.9 (13.4) |

Abbreviations; HPSF = Healthy Primary School of the Future, SD = standard deviation.
