# Supplementary material for: Food for thought? The effects of the Healthy Primary School of the Future on children’s educational outcomes
Source: PLoS One. 2026 Jun 24;21(6):e0334638. doi: 10.1371/journal.pone.0334638 (PMC13293421; doi:10.1371/journal.pone.0334638)
Supplement: S2 Fig — Changes in scores per year are compared with national averages derived from the Pupil Administration System (LAS) ParnasSys database [43]. (DOCX) [file pone.0334638.s002.docx]

**S2 Figure 2. Theoretical example of effect of full HPSF and control on mathematics**

The following numbers were based on observed values from a child on a control school, estimated mean differences between full HPSF and control (Table 2), and national averages calculated from Pupil Administration System (LAS) ParnasSys database [43]

**Control Group:**

- Change in scores: 17.5 (Year 1), 8.5 (Year 2), 12.5 (Year 3), 13.2 (Year 4)
- Average Increase: (17.5+8.5+12.5+13.2)/4​ = 12.9 points/year

**Full HPSF Group:**

- Change in scores: 23.7 (Year 1), 10.7 (Year 2), 12.0 (Year 3), 19.7 (Year 4)
- Average Increase: (23.7+10.7+12.0+19.7​)/4 = 16.5 points/year

**National Average:**

- Change in scores: 19.1 (Year 1), 17.4 (Year 2), 12.1 (Year 3), 13.2 (Year 4)
- Average Increase: (19.1+17.4+12.1+13.2​)/4 = 15.5 points/year

I
