# Supplementary material for: Food for thought? The effects of the Healthy Primary School of the Future on children’s educational outcomes
Source: PLoS One. 2026 Jun 24;21(6):e0334638. doi: 10.1371/journal.pone.0334638 (PMC13293421; doi:10.1371/journal.pone.0334638)
Supplement: S3 Fig — (DOCX) [file pone.0334638.s003.docx]

**S3 Figure 3. Mathematics performance change after one year compared to the national Dutch average**

**
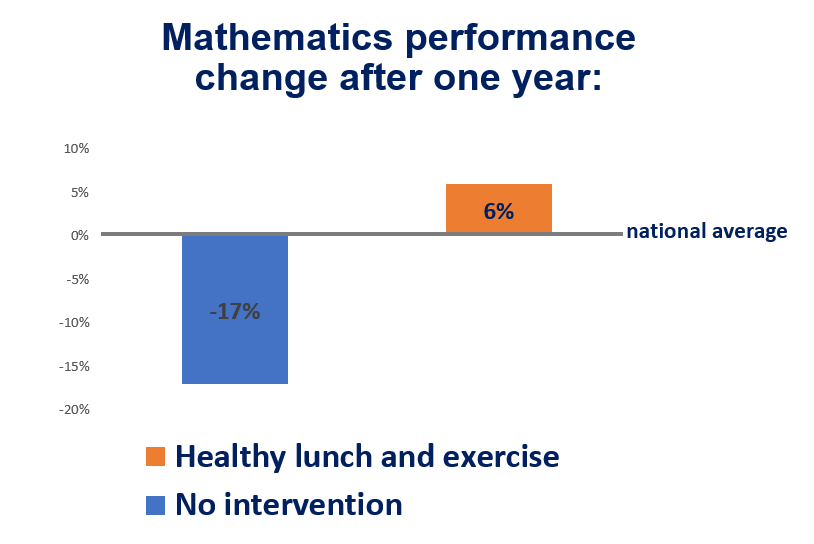
**
