# Supplementary material for: Food for thought? The effects of the Healthy Primary School of the Future on children’s educational outcomes
Source: PLoS One. 2026 Jun 24;21(6):e0334638. doi: 10.1371/journal.pone.0334638 (PMC13293421; doi:10.1371/journal.pone.0334638)
Supplement: S1 Text — Syntax file used for the analyses of mathematics and Dutch language outcomes. (DOCX) [file pone.0334638.s004.docx]

S1 Text. Syntax for mathematics and Dutch language analyses.

Syntax file used for the analyses of mathematics and Dutch language outcomes‌‌.

* Encoding: UTF-8.

*DATASET: 20240327_Rekenen_kort.sav intact.sav

USE ALL.

COMPUTE filter_$=(Jaartal>= 2016 & PrimaryLast = 1 & schooltype_mean < 4).

VARIABLE LABELS filter_$ 'Jaartal>= 2016 & & PrimaryLast = 1& schooltype_mean < 4 (FILTER)'.

VALUE LABELS filter_$ 0 'Not Selected' 1 'Selected'.

FORMATS filter_$ (f1.0).

FILTER BY filter_$.

EXECUTE.

*Opsplitsen naar interventiegroep.

SORT CASES BY Schooltype_mean.

SPLIT FILE LAYERED BY Schooltype_mean.

EXAMINE VARIABLES=Score_numeriek_mean BY Exposure

/PLOT BOXPLOT HISTOGRAM NPPLOT

/COMPARE GROUPS

/STATISTICS DESCRIPTIVES

/CINTERVAL 95

/MISSING LISTWISE

/NOTOTAL.

*Selecteer alleen de toetsen waarbij score bekend is.

USE ALL.

COMPUTE filter_$=(Jaartal>= 2016 & PrimaryLast = 1 & schooltype_mean < 4 & Score_numeriek_mean ~= 999).

VARIABLE LABELS filter_$ 'Jaartal>= 2016 & & PrimaryLast = 1& schooltype_mean < 4 & Score_numeriek_mean ~= 999 (FILTER)'.

VALUE LABELS filter_$ 0 'Not Selected' 1 'Selected'.

FORMATS filter_$ (f1.0).

FILTER BY filter_$.

EXECUTE.

FREQUENCIES VARIABLES=Versie DIGI

/ORDER=ANALYSIS.

*DATASET: BRINS2018-2019-kort - Nederlands - aggregate - 2024_08_22.sav

USE ALL.

COMPUTE filter_$=(Jaartal>= 2016 & PrimaryLast = 1 & schooltype_mean < 4).

VARIABLE LABELS filter_$ 'Jaartal>= 2016 & & PrimaryLast = 1& schooltype_mean < 4 (FILTER)'.

VALUE LABELS filter_$ 0 'Not Selected' 1 'Selected'.

FORMATS filter_$ (f1.0).

FILTER BY filter_$.

EXECUTE.

*Opsplitsen naar interventiegroep.

SORT CASES BY Schooltype_mean.

SPLIT FILE LAYERED BY Schooltype_mean.

EXAMINE VARIABLES=Score_numeriek_mean BY Exposure

/PLOT BOXPLOT HISTOGRAM NPPLOT

/COMPARE GROUPS

/STATISTICS DESCRIPTIVES

/CINTERVAL 95

/MISSING LISTWISE

/NOTOTAL.

*Selecteer alleen de toetsen waarbij score bekend is.

USE ALL.

COMPUTE filter_$=(Jaartal>= 2016 & PrimaryLast = 1 & schooltype_mean < 4 & Score_numeriek_mean ~= 999).

VARIABLE LABELS filter_$ 'Jaartal>= 2016 & & PrimaryLast = 1& schooltype_mean < 4 & Score_numeriek_mean ~= 999 (FILTER)'.

VALUE LABELS filter_$ 0 'Not Selected' 1 'Selected'.

FORMATS filter_$ (f1.0).

FILTER BY filter_$.

EXECUTE.

FREQUENCIES VARIABLES=Versie DIGI

/ORDER=ANALYSIS.
