# Supplementary material for: Food for thought? The effects of the Healthy Primary School of the Future on children’s educational outcomes
Source: PLoS One. 2026 Jun 24;21(6):e0334638. doi: 10.1371/journal.pone.0334638 (PMC13293421; doi:10.1371/journal.pone.0334638)
Supplement: S1 Table — Supplementary table presenting the full mixed model results for the intervention effects on mathematics and reading comprehension. (DOCX) [file pone.0334638.s005.docx]

**Supplementary Table 1:** Mixed model for repeated measures analysis – additional results

Overall F-test for interaction between group and exposure:

- Mathematics: F_6, 267.5_ = 5.110, p < 0.001
- Reading comprehension: F_6, 929.6_ = 5.838, p < 0.001

Variance-covariance matrix

| **Mathematics** | delta1 | delta2 | delta3 | delta4 |
| --- | --- | --- | --- | --- |
| delta1 | 437.7 |  |  |  |
| delta2 | 333.4 | 436.3 |  |  |
| delta3 | 301.4 | 371.3 | 457.2 |  |
| delta4 | 235.1 | 308.0 | 372.1 | 475.8 |
|  |  |  |  |  |
| **Reading Comprehension** | delta1 | delta2 | delta3 | delta4 |
| delta1 | 94.7 |  |  |  |
| delta2 | 56.3 | 98.0 |  |  |
| delta3 | 56.1 | 72.1 | 124.3 |  |
| delta4 | 77.0 | 76.6 | 106.2 | 165.0 |
