# Supplementary material for: Food for thought? The effects of the Healthy Primary School of the Future on children’s educational outcomes
Source: PLoS One. 2026 Jun 24;21(6):e0334638. doi: 10.1371/journal.pone.0334638 (PMC13293421; doi:10.1371/journal.pone.0334638)
Supplement: S2 Table — Supplementary table presenting participant flow and available test-score data across. (DOCX) [file pone.0334638.s006.docx]

**Supplementary Table 2**

Flowchart Participant flow across exposure waves

Children with baseline data

Reading comprehension: N = 4191 Mathematics: N = 3857

Exposure 1

Measured: 3872

Missing: 319

Exposure 2

Measured: 3741

Missing: 450

Exposure 3

Measured: 3625

Missing: 566

Exposure 4

Measured: 3498

Missing: 693

Exposure 1

Measured: 3580

Missing: 277

Exposure 2

Measured: 3462

Missing: 395

Exposure 3

Measured: 3351

Missing: 506

Exposure 4

Measured: 3224

Missing: 633
