# Supplementary material for: Food for thought? The effects of the Healthy Primary School of the Future on children’s educational outcomes
Source: PLoS One. 2026 Jun 24;21(6):e0334638. doi: 10.1371/journal.pone.0334638 (PMC13293421; doi:10.1371/journal.pone.0334638)

PLOS ONE

Food for Thought? The Effects of the Healthy Primary School of the Future on Children’s Educational Outcomes

--Manuscript Draft--

Manuscript Number:	PONE-D-24-56254
Article Type:	Research Article
Full Title:	Food for Thought? The Effects of the Healthy Primary School of the Future on Children’s Educational Outcomes
Short Title:	Food for Thought? The Effects of the Healthy Primary School of the Future on Children’s Educational Outcomes
Corresponding Author:	Bo Henriette Wilhelmina van Engelen, MSc<br>Maastricht University Faculty of Health, Medicine and Life Sciences: Maastricht Universitair Medisch Centrum+<br>Maastricht, NETHERLANDS, KINGDOM OF THE
Keywords:	*Primary school<br>*Health promoting school<br>*Educational performance<br>*Children
Abstract:	Background:<br>There is limited empirical evidence regarding the effects of school-based health-promoting interventions on educational outcomes, highlighting a need for further research in this area to understand their broader impact on academic performance. The Healthy Primary School of the Future (HPSF) is a Dutch intervention aimed at improving children’s health by providing healthy school lunches and structured physical activity (PA) sessions. While HPSF’s positive impact on physical health has been well-documented, its effects on academic outcomes, particularly in mathematics and reading comprehension, remain less understood. This study evaluated the influence of HPSF on children's performance by using national standardised tests for these academic domains.<br>Methods:<br>A longitudinal quasi-experimental design was employed involving eight Dutch primary schools over a four-year period. Schools included two full HPSF schools (implementing both PA and healthy lunches), two partial HPSF schools (PA only), and four control schools (all in the same region). Scores from biannually taken national standardised tests in mathematics and reading comprehension were combined within the same year to decrease the number of missing values. The data were analysed using marginal models for repeated measures to assess the intervention effects over time.<br>Results:<br>Children in full HPSF schools demonstrated significant improvements in mathematics performance compared to control schools (standardised effect size (ES) = 0.30, p = 0.011 after one year, increasing to ES = 0.66, p < 0.001 after four years). Mathematics gains in partial HPSF schools were smaller and non-significant (ES≤0.23, p≥0.137). For reading comprehension, small but significant improvement was observed in year 1 for full HPSF schools compared with control schools (ES = 0.28, p < 0.001), but this effect was diminished by year 4. Partial HPSF schools showed sustained small gains in reading comprehension over time (ES between 0.06 and 0.39).<br>Conclusions:<br>The HPSF intervention significantly enhanced mathematics performance when both healthy lunches and PA sessions were implemented, underlining the importance of a holistic approach to health promotion in schools. However, the intervention’s impact on reading comprehension was limited, indicating the need for additional and/or more targeted strategies to improve literacy outcomes. These findings highlight the potential of integrated health interventions to boost academic performance and address both health and educational inequities.
Order of Authors:	Bo Henriette Wilhelmina van Engelen, MSc
	Marla T.H. Hahnraths

Powered by Editorial Manager® and ProduXion Manager® from Aries Systems Corporation

	Bjorn W. Winkens
	Trudie Schils
	Onno C.P. van Schayck
**Additional Information:**	
**Question**	**Response**
**Financial Disclosure**<br><br>Enter a financial disclosure statement that describes the sources of funding for the work included in this submission. Review the submission guidelines for detailed requirements. View published research articles from *PLOS ONE* for specific examples.<br><br>This statement is required for submission and **will appear in the published article** if the submission is accepted. Please make sure it is accurate.<br><br>**Funded studies**<br>Enter a statement with the following details:<br>• Initials of the authors who received each award<br>• Grant numbers awarded to each author<br>• The full name of each funder<br>• URL of each funder website<br>• Did the sponsors or funders play any role in the study design, data collection and analysis, decision to publish, or preparation of the manuscript?<br><br>Did you receive funding for this work?	Yes
Please add funding details.<br>as follow-up to "**Financial Disclosure**<br><br>Enter a financial disclosure statement that describes the sources of funding for the work included in this submission. Review the submission guidelines for detailed requirements. View published research articles from *PLOS ONE* for specific examples.<br><br>This statement is required for submission and **will appear in the published article** if	De OnderwijsMonitor Limburg was funded by Kennisas project Educatieve Agenda Limburg, Provincial Government of Limburg, Maastricht University, and primary and secondary schools in the province of Limburg. The HPSF project was funded by the Limburg provincial authorities, Project Number 200130003, by Friesland Campina, Project Number LLMV00, and by Maastricht University, Project Number 200130003.

the submission is accepted. Please make sure it is accurate.<br><br>**Funded studies**<br>Enter a statement with the following details:<br>• Initials of the authors who received each award<br>• Grant numbers awarded to each author<br>• The full name of each funder<br>• URL of each funder website<br>• Did the sponsors or funders play any role in the study design, data collection and analysis, decision to publish, or preparation of the manuscript?<br><br>Did you receive funding for this work?"	
Please select the country of your main research funder (please select carefully as in some cases this is used in fee calculation).<br>as follow-up to "**Financial Disclosure**<br><br>Enter a financial disclosure statement that describes the sources of funding for the work included in this submission. Review the submission guidelines for detailed requirements. View published research articles from *PLOS ONE* for specific examples.<br><br>This statement is required for submission and **will appear in the published article** if the submission is accepted. Please make sure it is accurate.<br><br>**Funded studies**<br>Enter a statement with the following details:<br>• Initials of the authors who received each award<br>• Grant numbers awarded to each author<br>• The full name of each funder<br>• URL of each funder website<br>• Did the sponsors or funders play any role in the study design, data collection and analysis, decision to publish, or preparation of the manuscript?	NETHERLANDS - NL

Powered by Editorial Manager® and ProduXion Manager® from Aries Systems Corporation

Did you receive funding for this work?"	
**Competing Interests**<br><br>Use the instructions below to enter a competing interest statement for this submission. On behalf of all authors, disclose any competing interests that could be perceived to bias this work—acknowledging all financial support and any other relevant financial or non-financial competing interests.<br><br>This statement is required for submission and **will appear in the published article** if the submission is accepted. Please make sure it is accurate and that any funding sources listed in your Funding Information later in the submission form are also declared in your Financial Disclosure statement.<br><br>View published research articles from *PLOS ONE* for specific examples.<br><br>**NO authors have competing interests**<br><br>Enter: *The authors have declared that no competing interests exist.*<br><br>**Authors with competing interests**<br><br>Enter competing interest details beginning with this statement:<br><br>*I have read the journal's policy and the authors of this manuscript have the following competing interests: [insert competing interests here]*<br><br>* typeset	The authors declare that they have no competing interests.
**Ethics Statement**<br><br>Enter an ethics statement for this submission. This statement is required if the study involved:<br><br>• Human participants<br>• Human specimens or tissue	The need for Medical Ethical approval has been waived by the Zuyderland Medical Ethics Committee in Heerlen (METCZ:14N-142). Children were allowed to participate in the measurements if their parents or guardians signed an informed consent. Data on educational performance described in this paper stems from an already existing data infrastructure that is granted with ethical approval by the Ethical Review Committee Inner City faculties. Permission to collect the data was granted (ERCIC_092_12_07_2018).

• Vertebrate animals or cephalopods<br>• Vertebrate embryos or tissues<br>• Field research<br><br>Write "N/A" if the submission does not require an ethics statement.<br><br>General guidance is provided below. Consult the submission guidelines for detailed instructions. **Make sure that all information entered here is included in the Methods section of the manuscript.**	

Powered by Editorial Manager® and ProduXion Manager® from Aries Systems Corporation

**Format for specific study types**<br><br>**Human Subject Research (involving human participants and/or tissue)**<br>• Give the name of the institutional review board or ethics committee that approved the study<br>• Include the approval number and/or a statement indicating approval of this research<br>• Indicate the form of consent obtained (written/oral) or the reason that consent was not obtained (e.g. the data were analyzed anonymously)<br><br>**Animal Research (involving vertebrate animals, embryos or tissues)**<br>• Provide the name of the Institutional Animal Care and Use Committee (IACUC) or other relevant ethics board that reviewed the study protocol, and indicate whether they approved this research or granted a formal waiver of ethical approval<br>• Include an approval number if one was obtained<br>• If the study involved *non-human primates*, add *additional details* about animal welfare and steps taken to ameliorate suffering<br>• If anesthesia, euthanasia, or any kind of animal sacrifice is part of the study, include briefly which substances and/or methods were applied<br><br>**Field Research**<br><br>Include the following details if this study involves the collection of plant, animal, or other materials from a natural setting:<br>• Field permit number<br>• Name of the institution or relevant body that granted permission	
**Data Availability**<br><br>Authors are required to make all data underlying the findings described fully available, without restriction, and from the time of publication. PLOS allows rare exceptions to address legal and ethical concerns. See the PLOS Data Policy and FAQ for detailed information.	Yes - all data are fully available without restriction

A Data Availability Statement describing where the data can be found is required at submission. Your answers to this question constitute the Data Availability Statement and **will be published in the article**, if accepted.<br><br>**Important:** Stating 'data available on request from the author' is not sufficient. If your data are only available upon request, select 'No' for the first question and explain your exceptional situation in the text box.<br><br>Do the authors confirm that all data underlying the findings described in their manuscript are fully available without restriction?	
**Describe where the data may be found in full sentences. If you are copying our sample text, replace any instances of XXX with the appropriate details.**<br><br>• If the data are **held or will be held in a public repository**, include URLs, accession numbers or DOIs. If this information will only be available after acceptance, indicate this by ticking the box below. For example: *All XXX files are available from the XXX database (accession number(s) XXX, XXX.).*<br>• If the data are all contained **within the manuscript and/or Supporting Information files**, enter the following: *All relevant data are within the manuscript and its Supporting Information files.*<br>• If neither of these applies but you are able to provide **details of access elsewhere**, with or without limitations, please do so. For example:<br><br>*Data cannot be shared publicly because of [XXX]. Data are available from the XXX Institutional Data Access / Ethics Committee (contact via XXX) for researchers who meet the criteria for access to confidential data.*<br><br>*The data underlying the results presented in the study are available from (include the name of the third party*	All relevant data are within the manuscript and its Supporting Information files.

and contact information or URL).<br>• This text is appropriate if the data are owned by a third party and authors do not have permission to share the data.<br><br>* typeset	
Additional data availability information:	

Powered by Editorial Manager® and ProduXion Manager® from Aries Systems Corporation

Manuscript

Click here to access/download;Manuscript;Food for Thought 19122024.docx

Food for Thought? The Effects of the Healthy Primary School of the Future on Children's Educational Outcomes

Bo H. W. van Engelen [a] *, Marla T. H. Hahnraths [a], Bjorn Winkens [b], Trudie Schils [c] and Onno C. P. van Schayck [a]

[a] Department of Family Medicine, Care and Public Health Research Institute (CAPHRI), Maastricht University, P.O. Box 616, 6200 MD, Maastricht, The Netherlands; b.vanengelen@maastrichtuniversity.nl (B.H.W.v.E.) mth.hahnraths@maastrichtuniversity.nl (M.T.H.H.), onno.vanschayck@maastrichtuniversity.nl (O.C.P.v.S.)

[b] Department of Methodology and Statistics, Care and Public Health Research Institute (CAPHRI), Maastricht University, P.O. Box 616, 6200 MD Maastricht, The Netherlands; bjorn.winkens@maastrichtuniversity.nl (B.W.)

[c] Department of Macro, International & Labour Economics, School of Business and Economics, Maastricht University, P.O. Box 616, 6200 MD Maastricht, The Netherlands;

* Correspondence: b.vanengelen@maastrichtuniversity.nl; Tel.: +31 43 388 7066

Abstract

Background:
There is limited empirical evidence regarding the effects of school-based health-promoting interventions on educational outcomes, highlighting a need for further research in this area to understand their broader impact on academic performance. The Healthy Primary School of the Future (HPSF) is a Dutch intervention aimed at improving children's health by providing healthy school lunches and structured physical activity (PA) sessions. While HPSF's positive impact on physical health has been well-documented, its effects on academic outcomes, particularly in mathematics and reading comprehension, remain less understood. This study evaluated the influence of HPSF on children's performance by using national standardised tests for these academic domains.

Methods:
A longitudinal quasi-experimental design was employed involving eight Dutch primary schools over a four-year period. Schools included two full HPSF schools (implementing both PA and healthy lunches), two partial HPSF schools (PA only), and four control schools (all in the same region). Scores from biannually taken national standardised tests in mathematics and reading comprehension were combined within the same year to decrease the number of missing values. The data were analysed using marginal models for repeated measures to assess the intervention effects over time.

Results:
Children in full HPSF schools demonstrated significant improvements in mathematics performance compared to control schools (standardised effect size (ES) = 0.30, $p = 0.011$ after one year, increasing to ES = 0.66, $p < 0.001$ after four years). Mathematics gains in partial HPSF schools were smaller and non-significant (ES≤0.23, p≥0.137). For reading comprehension, small but significant improvement was observed in year 1 for full HPSF schools compared with control schools (ES = 0.28, $p < 0.001$), but this effect was diminished by year 4. Partial HPSF schools showed sustained small gains in reading comprehension over time (ES between 0.06 and 0.39).

Conclusions:
The HPSF intervention significantly enhanced mathematics performance when both healthy lunches and PA sessions were implemented, underlining the importance of a holistic approach to health promotion in schools. However, the intervention's impact on reading comprehension was limited, indicating the need for additional and/or more targeted strategies to improve literacy outcomes. These findings highlight the potential of integrated health interventions to boost academic performance and address both health and educational inequities.

Key words

- Primary school
- Health promoting school
- Educational performance
- Children

1. Introduction

Several studies show that school performance of Dutch children has been declining in recent years and nationally set target levels are not met [1]. Both reading skills of primary school children aged 9-10 years and 11-12 years declined compared to earlier cohorts [1,2]. National goals are that at the end of primary school, at least 85 percent of the children reach a basic or fundamental level of reading and language skills and at least 65 percent reaches a target level for these skills. While over 90 percent of the children met the basic level between 2015 and 2022, the target level is not met in all domains of language skills [3-6]. Similar, for mathematics the majority of Dutch primary school children currently do not achieve both the basic and the target level.

Over the last years, children and adolescents are often the focus in health research [7]. As lifestyle behaviours formed during childhood are likely to track into adulthood, health promotion during childhood could not only lead to short-term health benefits for the child, but could ultimately also lead to a healthier adult population [8-10]. This idea, together with the notion that the health status of children in Western countries (including the Netherlands) is currently suboptimal across various domains (e.g., weight status, physical activity (PA), and dietary habits) [11-13] has led to the development, implementation, and evaluation of various health-promoting interventions targeting children. These interventions are often implemented in influential settings such as the school setting, where children spend a considerable amount of their time for several critical developmental years [14,15].

The effects of school-based health-promoting interventions have been investigated on a wide range of health outcomes such as weight status and dietary and PA behaviours [16-20]. However, evidence on the effects of school-based health promotion on children's educational outcomes is limited [14], despite the strong association between children's health and their learning capacity [21]. In the study of Schwartz and Rothbart, where free school lunches were provided, a significant improvement in academic outcomes, particularly in mathematics and reading test scores was observed [22]. Additionally, the study by Asigbee et al. (2018) revealed that higher PA levels and proper nutrition were significantly associated with improved academic performance, with students in the healthy nutrition group scoring higher on standardised tests in reading, mathematics, and science [23].

The Healthy Primary School of the Future (HPSF) is a Dutch intervention aiming to integrate health in the primary school system [24]. Between 2015-2019, the effects of HPSF were investigated in an efficacy trial involving eight Dutch primary schools: two full intervention schools, where a daily healthy school lunch was provided and structured PA sessions during the school day were incorporated, two partial intervention schools, where only the structured PA sessions were offered, and four control schools, which continued with their regular curriculum that is common practice in the Netherlands [24]. Analyses revealed significant positive intervention

effects on children's body mass index (BMI) z-score, waist circumference, and dietary and PA behaviours [25-27]. However, it remained unknown what the intervention's effects were on children's educational outcomes. Considering HPSF's positive impact on children's health and the strong link between children's health and their learning capacity [21], it was hypothesised that children from schools implementing HPSF might have better educational outcomes than children from primary schools not implementing HPSF.

Using the results from national standardised tests [28], the present study aimed to answer the following research questions:

- What is the effect of the daily provision of a healthy school lunch combined with structured PA sessions (full HPSF), compared with PA sessions alone (partial HPSF), versus no intervention (control), on children's performance in national standardised mathematics tests in primary schools?
- What is the effect of healthy lunch sessions combined with PA sessions (full HPSF), compared to PA sessions alone (partial HPSF), versus no intervention (control), on children's performance in national standardised reading comprehension tests in primary schools?

2. Methods

2.1 Study Design

The study had a longitudinal quasi-experimental design and involved eight Dutch primary schools: two full intervention schools (full HPSFs), two partial intervention schools (partial HPSFs), and four control schools. These schools were all recruited to participate in the HPSF four-year efficacy trial based on voluntary participation [24]. The implementation of the HPSF started in November 2015 and annual measurements were performed yearly up and until 2019. The main outcome parameters were student's scores on national standardised tests in mathematics and reading comprehension. These tests are administered to all students twice a year, normally in the middle of the school year in January/February and at the end of the school year in June.

2.2 The Healthy Primary School of the Future

HPSF was an intervention developed by the schoolboard of the participating schools, the regional public health services, and Maastricht University with the aim to sustainably integrate health in the school system [24]. The intervention consisted of two main changes: 1) daily provision of a free healthy school lunch, and 2) daily structured 60-minute PA sessions after lunch.

In all intervention schools, the school day was extended, which provided the opportunity to prolong the lunch break time with 45 to 75 min. In full HPSFs, a healthy school lunch was provided to all students, which is in sharp contrast with the normal situation in the Netherlands where students need to bring their lunch from home or go home to eat lunch. The school lunch was developed by a dietician, provided by catering services, and varied every ten weeks. At least 80% of the provided products met the dietary guidelines of the Dutch Health Council [29]. The lunch was provided in buffet style, allowing children to choose from various available food products. Children in both the full and partial HPSFs participated in structured PA sessions during lunch break time. These sessions were guided by teachers, pedagogical staff, and/or members of local sports clubs. Control schools continued with their regular curriculum that is common practice in the Netherlands.

The need for Medical Ethical approval has been waived by the Zuyderland Medical Ethics Committee in Heerlen (METCZ:14N-142). Children were allowed to participate in the measurements if their parents or guardians signed an informed consent.

2.3 Study Population

The schools included in the current study were member of one schoolboard and were located in the Parkstad region in the southern part of the Netherlands. This region is known as a low to moderate socioeconomic area that is characterised by a relatively high prevalence of chronic diseases and a low life expectancy, as compared with the rest of the Netherlands [30,31].

All students from study years 3-8 (aged 6-12 years; internationally comparable to grades 1-6) of the eight schools were included in the present study, as the Dutch standardised test system for all core parts of the curriculum (e.g., mathematics and reading comprehension) starts from study year 3.

2.4 Data Collection Procedures

In the Netherlands, all primary school students participate in national standardised tests. The tests are designed by CITO, a Dutch institute for test development (Central Institute for Test Development) and are advised to be administered twice a year (mid-school year (M); January/February and end-of-school year (E); May/June) to track children's performance on a wide range of educational domains, including reading comprehension and mathematics [1]. Teachers grade the tests using a standardised grading scheme which allows comparison of a student's results throughout their primary school time.

Students' test scores were retrieved through a regional monitor that is part of an ongoing research-practice-partnership between Maastricht University and primary and secondary schools in the province of Limburg: de Onderwijsmonitor Limburg. According to the passive consent principle, parents can withdraw their child anytime.

2.5 Measures

The main outcome variables of the present study are students' standardised test scores on the domains mathematics and reading comprehension, as these domains are likely most prone to be influenced by external factors such as the school environment and are a good representation of general knowledge (Inspectie van het Onderwijs, 2024). In the current study, the mid-school year (M) and end-of-school year (E) standardised test scores of each student were combined by calculating an average score from the M and E tests of the same year, for each time point. If one of these test results was missing, we used the remaining available test score from that time point. However, if both test results were unavailable, the data for that time point were marked as missing. During study duration, two test versions (version A and version B), which do not differ on content but on outcome scale, as well as two entry methods of the tests (digital or on paper) were used, which we accounted for in the data analyses.

2.6 Data Analysis

Analyses were performed using IBM SPSS Statistics for Windows (version 29.0.2.0, Armonk, NY: IBM Corp). Figures were created using the forestplot package within Rstudio (version 2023.12.1+402) and R (version 4.3.3). Categorical variables were presented by number and percentage, whereas means and standard deviations (SD) were used for numerical variables. Pearson chi-square tests were used to compare proportions, while one-way ANOVA was applied to compare mean values. If homoscedasticity assumption (based on Levene's test) was violated, Welch tests for overall comparison and Games-Howell tests for pairwise comparisons were used.

For the scores on mathematics and reading comprehension, the number of students for whom data were available and the observed mean and standard deviation (SD) per intervention group (full HPSF, partial HPSF, control) after one, two, three, and four years of exposure to HPSF were computed. To assess the intervention effects at the different timepoints, a marginal model for repeated measures with intervention group (full HPSF, partial HPSF, control), exposure (1, 2, 3, 4 years), and their interaction as well as test version (version A or B), age (in years), and sex (male/female) as independent variables was used, where an unstructured covariance structure for repeated measurements was used. For mathematics, the standardised test score measured at baseline (average of

the two years preceding the start of HPSF) was added to the model to correct for baseline differences. For reading comprehension, this baseline score was missing for almost all students and therefore not included in the model. The entry method of the tests (digital or on paper) was not included as a fixed factor, since there were only a few tests made on paper for mathematics, while for reading comprehension, version A was mainly digital and version B was mainly made on paper. The marginal model for repeated measures was used because it included all available data and assumed missing values to be at random (MAR), using a restricted maximum likelihood-based approach for estimation. Corrected mean differences after one, two, three, and four years of exposure (delta1-delta4) with their corresponding 95% confidence intervals (CI) are presented. Standardised effect sizes (ES) were computed as the corrected mean difference at each timepoint (year of exposure) divided by the square root of the residual variance at the corresponding year of exposure, where ES=0.2 is considered a small effect, 0.5 a medium effect, and 0.8 a large effect [33] (Cohen, 1988). Two-sided p-values ≤ 0.05 were considered statistically significant. Additionally, a Bonferroni approach was considered to account for multiple group comparisons (significance level $\alpha = 0.05/3 = 0.0167$).

3. Results

In total, 4191 mathematics tests and 3857 reading comprehension tests were included in the current study. For mathematics, the majority of tests was made using test version B (full HPSF 1199, 90.3%; partial HPSF 537, 65.4%; and control 1629, 79.8%). The same applied to the reading comprehension tests (full HPSF 3094, 80.2%; partial HPSF 1189, 90.6 %; and control 1436, 80%). Furthermore, a very small proportion of the mathematics tests was made on paper (full HPSF 2, 0.4%; partial HPSF 9, 0.2%; and control 7, 0.3%). For reading comprehension, most tests were made on paper (full HPSF 1191, 90.7%; partial HPSF 469, 62.7%; and control 1439, 80.1%) (Table 1).

As for sex and age of participants, the proportion of female children was close to 50% in each group (50.7% in full HPSF, 44.6% in partial HPSF, 51.3% in control; Pearson chi-square test $p = 0.127$), while there was a significant difference in age among the three intervention groups (Welch test $p = 0.006$). Pairwise comparisons showed that the children in the partial HPSF group were significantly younger (n=307, mean age = 6.9 years, SD = 0.8) than those in the full HPSF group (n=509, mean = 7.1, SD = 1.0, Games-Howell test $p = 0.015$) and in control schools (n = 786, mean = 7.1, SD = 0.9, Games-Howell test $p = 0.009$).

Table 1. Test characteristics.

	Total		Full HPSF		Partial HPSF		Control	
	**No. tests**	**No. tests (%)**	**No. tests**	**No. tests (%)**	**No. tests**	**No. tests (%)**	**No. tests**	**No. tests (%)**
**Mathematics**<br>Test version<br>*A*	4191	826 (19.7)	1328	129 (9.7)	821	284 (34.6)	2042	413 (20.2)
B		3365 (80.3)		1199 (90.3)		537 (65.4)		1629 (79.8)
Entry method<br>*Paper*	4191	18 (0.4)	1328	2 (0.2)	821	9 (1.1)	2042	7 (0.3)
Digital		4173 (99.6)		1326 (99.8)		812 (98.9)		2035 (99.7)
**Reading comprehension**<br>Test version<br>*A*	3857	763 (19.8)	1313	124 (9.4)	748	279 (37.3)	1796	360 (20.0)
B		3094 (80.2)		1189 (90.6)		469 (62.7)		1436 (80.0)
Entry method<br>*Paper*	3857	3099 (80.3)	1313	1191 (90.7)	748	469 (62.7)	1796	1439 (80.1)
Digital		758 (19.7)		122 (9.3)		279 (37.3)		357 (19.9)

Abbreviations; No. = number, HPSF = Healthy Primary School of the Future.

3.1 Intervention Effects

The full HPSF group showed a significant improvement in mean mathematics score compared to the control group in the first year (corrected mean difference = 6.2, 95%CI 1.4 to 10.9, $p = 0.011$), corresponding to a small-to-medium ES of 0.30. This effect remained significant (all $p \leq 0.001$) for the following years, with ES ranging from 0.37 to 0.66 (Table 2 and Figure 1a). The full HPSF group also showed a significant improvement in mathematics score compared to the partial HPSF group in years 2 to 4 (all $p \leq 0.006$) with medium to large ES (0.47, 0.43, and 0.86 for years 2, 3, and 4, respectively) (Table 2 and Figure 1b). The partial group showed no significant improvement in mathematics score compared to the control group (Table 2 and Figure 1c).

As for reading comprehension, the full HPSF group showed only a significant intervention effect compared to the control group in year 1 (corrected mean difference = 2.7, 95%CI 1.2 to 4.2, $p < 0.001$), which corresponded to an ES of 0.28 (Table 2 and Figure 2a). The full HPSF group had a significantly lower mean reading comprehension score than the partial HPSF group after 4 years (corrected mean difference = -3.5, 95%CI -5.8, -1.2, $p = 0.003$) with a small-to-medium ES of 0.27 (Table 2 and Figure 2b). In addition, the partial HPSF group

scored significantly higher than the control group in year 1, 2, and 4 (all p ≤ 0.003) with small-to-medium ES of 0.28, 0.24, and 0.39, respectively (Table 2 and Figure 2c).

For the observed mean test scores for mathematics and reading comprehension and standard deviations for each intervention group over time, see Appendix Table 1.

Table 2. Estimated intervention effects (corrected mean differences (B), 95% confidence intervals, two-sided p-values, and ES) after one, two, three and four years of exposure (delta1-delta4) with correction for baseline (mathematics) and age, sex, and test version (mathematics and reading comprehension).

		**Full HPSF vs. control**			**Full HPSF vs. partial HPSF**			**Partial HPSF vs. control**		
		**B (95% CI)**	***p***	**ES**	**B (95% CI)**	***p***	**ES**	**B (95% CI)**	***p***	**ES**
**Mathematics**	**delta1**	6.182 (1.418, 10.945)	**0.011**	0.30	1.449 (-5.098, 7.996)	0.664	0.07	4.732 (-1.512, 10.977)	0.137	0.23
	**delta2**	8.434 (3.689, 13.178)	**<0.001**	0.40	9.756 (3.233, 16.279)	**0.003**	0.47	-1.323 (-7.532, 4.887)	0.676	0.06
	**delta3**	7.948 (3.104, 12.792)	**0.001**	0.37	9.208 (2.628, 15.789)	**0.006**	0.43	-1.260 (-7.552, 5.032)	0.694	0.06
	**delta4**	14.425 (8.448, 20.403)	**<0.001**	0.66	18.713 (11.057, 26.369)	**<0.001**	0.86	-4.287 (-11.646, 3.072)	0.253	0.20
**Reading Comprehension**	**delta1**	2.690 (1.176, 4.206)	**<0.001**	0.28	-0.012 (-1.933, 1.910)	0.991	0.001	2.701 (0.895, 4.508)	**0.003**	0.28
	**delta2**	0.934 (-0.300,2.169)	0.138	0.09	-1.401 (-2.986, 0.183)	0.083	0.14	2.336 (0.800, 3.872)	**0.003**	0.24
	**delta3**	0.670 (-0.691, 2.031)	0.334	0.06	-0.037 (-1.822, 1.748)	0.968	0.003	0.707 (-0.965, 2.379)	0.407	0.06
	**delta4**	1.511 (-0.246, 3.267)	0.092	0.12	-3.507 (-5.780, -1.233)	**0.003**	0.27	5.017 (2.893, 7.141)	**<0.001**	0.39

Abbreviations; HPSF = Healthy Primary School of the Future, CI = confidence interval, ES = effect size.

Figure 1: *Mathematics* – estimated intervention effect, i.e., difference in estimated means after one, two, three, and four years of exposure (delta1-delta4) corrected for outcome measured at baseline, test version, age, and sex. a) Full HPSF vs. control, b) Full HPSF vs. Partial HPSF, c) Partial HPSF vs. control. Abbreviations: HPSF = Healthy Primary School of the Future, CI = confidence interval.

Insert Figure 1a, 1b and 1c

Figure 2: *Reading Comprehension* – estimated intervention effect, i.e., difference in estimated means after one, two, three, and four years of exposure (delta1-delta4) corrected for test version, age, and sex. a) Full HPSF vs. control, b) Full HPSF vs. Partial HPSF, c) Partial HPSF vs. control. Abbreviations: HPSF = Healthy Primary School of the Future, CI = confidence interval.

Insert figure 2a,2b and 2c

4. Discussion

The present study explored the impact of the HPSF intervention on children's educational outcomes, specifically focusing on mathematics and reading comprehension performance.

4.1 Mathematics Performance

The outcomes in this study indicate that the full HPSF intervention led to significantly improved mathematics performance compared to control schools. After one year of exposure, the full HPSF group outperformed the control group (ES = 0.30, $p = 0.011$), and this improvement persisted and even grew stronger over time, with the highest ES observed after four years of exposure (ES = 0.66, $p < 0.001$). There was no significant effect of the partial HPSF group versus the control group, underpinning the importance of the combination of a healthy school lunch and structured PA sessions.

To put the effect of full HPSF versus control on mathematics score in perspective, we used the observed data from a child on a control school to compute the annual increase over time and translate this to a theoretical annual increase in case this child was on a full HPSF and additionally compare this to the national average annual increase in the same period (Appendix II). From this theoretical example we can see that the child in the control group demonstrated a modest average increase of 12.9 points per year, reflecting individual progress augmented by national trends. In contrast, based on the estimated full intervention effects, this child would have a higher average annual increase of 16.5 points per year if this child went to a HPSF. Interestingly, the national average increase across the same period was 15.5 points per year, indicating that while the full HPSF group outperformed the national trend with approximately 6%, the control group fell short of it with approximately 17% (Appendix II).

.

Research has consistently shown that PA can enhance cognitive functions such as executive function and memory, which are critical for learning tasks, including mathematics [33.34]. Results of a meta-analysis by Muntaner-Mas et al. showed that PA was associated with significant improvements in mathematics test scores [35]. Furthermore, previous research showed that children with healthier dietary and PA behaviours performed better on standardized tests, including mathematics, while those with unhealthy diets and low activity levels scored lower in reading, mathematics, and science, even when controlling for socioeconomic status (SES), age, and sex [23]. HPSF's positive impact on children's dietary behaviours has already been demonstrated [25]. Potentially, this change in dietary behaviours (in combination with increased PA) consequently positively impacted children's concentration and cognitive functioning during academic tasks [36,37].

4.2 Reading Comprehension

In contrast to the clear improvements observed for mathematics performance, the effect of HPSF on reading comprehension was modest. After one year of exposure, a small but significant improvement was observed in the full HPSF group compared to the control group (ES = 0.28, $p < 0.001$), but the effect did not sustain over time. By the fourth year, the difference between the full HPSF group and the control group was no longer statistically significant (ES = 0.12, $p = 0.092$). There was a significant difference in mean reading comprehension scores between the partial HPSF group and the control group after one, two, and four years of exposure. However, the ES (0.06 – 0.39) were only ranging from very small to small-to-medium [32] and the significant difference was absent at year 3.

One potential explanation for the limited effect of HPSF on reading comprehension is that this domain may be less sensitive to changes in physical health and/or nutritional status compared to mathematics performance and more depending on a broad range of external factors whilst mathematics development is more depending on the school context. For language outcomes, data from three studies demonstrated a small, positive effect of PA notably in reading, although comprehension tasks showed no significant gains [35]. Previous research has suggested that while PA and diet can positively influence general cognitive functions, specific academic domains may be influenced by additional factors, such as language exposure and literacy-related activities outside of school [33,38]. Maybe home environment (even preceding to school environment) has already led to literacy development, leaving less room for direct improvement than for mathematical development [39]. It is also possible that the benefits of health promotion on reading skills take longer to manifest, or that reading comprehension improvements require more targeted educational interventions that were not addressed by HPSF.

4.3 Strengths and Limitations

A major strength of this study is its longitudinal quasi-experimental design, which allowed for the evaluation of HPSF's impact over a four-year period. The use of national standardised test scores, administered biannually, provided robust and reliable measures of educational outcomes. Additionally, the inclusion of both full and partial HPSF schools enabled a deeper understanding of the contribution of the combination of healthy school lunches and PA sessions to educational outcomes, as well as the specific contribution of PA sessions in isolation.

However, the study also had limitations. First, as previously indicated, the schools that participated in the current study were located in a low- to moderate socioeconomic region, which may limit the generalisability of the findings to other contexts. Second, as the present study did not include an intervention group only receiving a healthy school lunch, we cannot report on the impact of a healthy school lunch in isolation. This limits our ability to draw firm conclusions in this regard. Third, the study did not account for potential confounding factors such as SES, parental involvement, home learning environment, or extracurricular activities, all of which may influence academic performance [40].

In the current study, no control for SES was applied. Data about parental education had to be retrieved from parental surveys and contained about 50% missing values and therefore the risk of a potential selection bias was large. Parents with a low SES, especially those with a non-Dutch language background, can be less eager (or able) to complete the parental survey [30]. Dutch schools do not register the parental education level in their school administration. However, all schools included in the current study were member of one schoolboard and were located in the same region, which is known as a low to moderate socioeconomic area. Additionally, the study of Boudewijns et al. (2019) looked at the characteristics of the participants and found that these were comparable to regional averages in terms of SES, sex, and health characteristics, with no substantial lifestyle differences observed [30].

Furthermore, the outcomes for reading comprehension were not corrected for the outcome measured at baseline (due to too many missing values) so we cannot draw an unequivocal conclusion. Lastly, while the study focused on mathematics performance and reading comprehension, other important domains such as language skills and social-emotional development were not assessed.

4.4 Implications for Education, Health Promotion, and Future Research

The results of this study can have important implications for educational policy, health promotion in schools, and future research. First, they highlight the potential of integrating health-promoting activities, such as providing healthy school lunches and encouraging PA, to not only improve children's health but also to enhance academic outcomes, particularly with regard to their mathematics performance. These findings support the idea that a holistic approach to health and education can benefit multiple aspects of a child's development [41]. As far as we know this is the first controlled study performed in this specific area and therefore, we advise replication of the study in other SES environments, as well in other countries, before more general conclusions may be drawn.

The study also revealed that the benefits of HPSF are not uniform across all academic domains, with stronger effects observed in mathematics compared to reading comprehension. This suggests that future interventions could benefit from tailoring to target specific educational outcomes more effectively.

Equity in education at primary school is an important issue in education, where it is aimed that all students are given equal opportunities to discover and develop their talents, regardless of their SES, ethnicity, or other circumstances. However, differences in learning achievements and educational opportunities between children from different backgrounds are visible in the Netherlands. Factors such as home situation, school quality, and early selection in education play a role [30]. The results of this research can contribute to equality of opportunity for all primary school students, as providing a healthy school lunch and more PA may improve learning and scholastic behaviour.

Further research exploring how (healthy) nutrition and PA influence academic outcomes across different settings—such as informal education and home learning environments—could contribute to a broader understanding of the mechanisms at work. Furthermore, more research focusing solely on nutritional interventions would allow to untangle the effects of dietary factors from other variables, offering a clearer understanding of how nutrition alone can impact cognitive functioning and academic performance.

4.5 Conclusion

In conclusion, the HPSF intervention demonstrated a strong positive influence on children's mathematics performance when the full HPSF intervention (i.e., both healthy lunches and structured PA sessions) was implemented. The intervention effects on reading comprehension were only present for the partial HPSF group, but these were relatively small and indecisive. The overall findings support the integration of health-promoting

interventions in primary schools. Through these interventions, both physical and academic development can be enhanced, which in turn can promote health and educational equity.

Acknowledgements

We thank all members of the research team for the work executed on this project. We thank Andrew Simons for providing the national average scores.

Human Subjects Approval Statement

Data on educational performance described in this paper stems from an already existing data infrastructure that is granted with ethical approval by the Ethical Review Committee Inner City faculties. Permission to collect the data was granted (ERCIC_092_12_07_2018).

Funding

De OnderwijsMonitor Limburg was funded by Kennisas project Educatieve Agenda Limburg, Provincial Government of Limburg, Maastricht University, and primary and secondary schools in the province of Limburg. The HPSF project was funded by the Limburg provincial authorities, Project Number 200130003, by Friesland Campina, Project Number LLMV00, and by Maastricht University, Project Number 200130003.

Conflict of Interest Disclosure Statement

The authors declare that they have no competing interests.

Authors' Contributions

Conceptualisation, BvE, MH, and OvS. Methodology, BvE, MH, and OvS. Analysis, BvE, MH, and BW. Investigation, BvE, MH, and BW. Data curation, BvE, MH, and BW. Writing – Original draft preparation, BvE. Writing – Review and editing, TS, MH, BW, and OvS. Supervision, BW and OvS. Project administration, BvE. Funding Acquisition, TS and OvS. All authors approved the final version for submission and agreed to be personally accountable for their own contributions and for ensuring that questions related to the accuracy or integrity of any part of the work, even ones in which the author was not personally involved, are appropriately investigated, resolved and documented in the literature.

References

1. Inspectie van het Onderwijs. *De Staat van het Onderwijs 2022: Trends en Ontwikkelingen* [Internet]. Utrecht: Inspectie van het Onderwijs; 2022 [cited 2024 Dec 3]. Only available in Dutch. Available from: https://www.onderwijsinspectie.nl/documenten/rapporten/2022/04/13/rapport-de-staat-van-het-onderwijs-2022
2. Swart NM, Gubbels JC, Zandt MP, Wolbers MH, Segers PC. PIRLS-2021: Trends in leesprestaties, leesattitude en leesgedrag van tienjarigen uit Nederland.
3. Inspectie van het Onderwijs. *The State of Education in the Netherlands 2015/2016* [Internet]. Utrecht: Inspectie van het Onderwijs; 2016 [cited 2024 Dec 3]. Available from: https://www.onderwijsinspectie.nl/documenten/rapporten/2016/04/13/the-state-of-education-in-the-netherlands-2015-2016
4. Inspectie van het Onderwijs. *The State of Education in the Netherlands 2016/2017* [Internet]. Utrecht: Inspectie van het Onderwijs; 2017 [cited 2024 Dec 3]. Available from: https://www.onderwijsinspectie.nl/documenten/rapporten/2017/04/12/the-state-of-education-in-the-netherlands-2016-2017
5. Inspectie van het Onderwijs. *The State of Education in the Netherlands 2019* [Internet]. Utrecht: Inspectie van het Onderwijs; 2019 [cited 2024 Dec 3]. Available from: https://www.onderwijsinspectie.nl/documenten/rapporten/2019/04/10/the-state-of-education-in-the-netherlands-2019
6. Inspectie van het Onderwijs. *The State of Education 2022* [Internet]. Utrecht: Inspectie van het Onderwijs; 2022 [cited 2024 Dec 3]. Available from: https://www.onderwijsinspectie.nl/documenten/rapporten/2022/04/13/the-state-of-education-2022
7. Patton GC, Sawyer SM, Santelli JS, Ross DA, Afifi R, Allen NB, et al. Our future: a Lancet commission on adolescent health and wellbeing [Internet]. Vol. 387, The Lancet. Lancet Publishing Group; 2016 [cited 2023 Dec 7]. p. 2423–78. Available from: http://dx.doi.org/10.1016/
8. Craigie AM, Lake AA, Kelly SA, Adamson AJ, Mathers JC. Tracking of obesity-related behaviours from childhood to adulthood: A systematic review. Vol. 70, Maturitas. Elsevier; 2011. p. 266–84.
9. Goldfield GS, Harvey A, Grattan K, Adamo KB. Physical Activity Promotion in the Preschool Years: A Critical Period to Intervene. Int J Environ Res Public Health [Internet]. 2012 Apr 16 [cited 2021 Feb 26];9(4):1326–42. Available from: http://www.mdpi.com/1660-4601/9/4/1326
10. Yang X, Telama R, Hirvensalo M, Tammelin T, Viikari JSA, Raitakari OT. Active commuting from

youth to adulthood and as a predictor of physical activity in early midlife: The Young Finns Study. Prev Med (Baltim). 2014 Feb 1;59(1):5–11.

11. CBS, RIVM, The Netherlands Nutrition Centre. Kinderen Eten te Weinig Fruit, Groente en vis [Children Do Not Consume Enough Fruit, Vegetables and Fish] [Internet]. 2017 [cited 2019 Aug 23]. Available from: https://www.cbs.nl/nl-nl/nieuws/2017/46/kinderen-eten-te-weinig-fruit-groente-en-vis
12. Volksgezondheidenzorg.info. Beweeggedrag Kinderen [Physical Activity Behaviour of Children]. [Internet]. [cited 2021 Feb 19]. Available from: https://www.volksgezondheidenzorg.info/onderwerp/sport-en-bewegen/cijfers-context/huidigesituatie#node-beweeggedrag-kinderen
13. Schönbeck Y, Talma H, von Dommelen P, Bakker B, Buitendijk SE, HiraSing RA, et al. Increase in prevalence of overweight in Dutch children and adolescents: A comparison of nationwide growth studies in 1980, 1997 and 2009. PLoS One. 2011;6(11):e27608.
14. Langford R, Bonell CP, Jones HE, Pouliou T, Murphy SM, Waters E, et al. The WHO Health Promoting School framework for improving the health and well-being of students and their academic achievement. Cochrane Database Syst Rev. 2014;2014(4).
15. FAO. Nutrition Education in Primary Schools [Internet]. Vol. 1. 2005. 64 p. Available from: http://www.fao.org/3/a-a0333e.pdf
16. Verrotti A, Penta L, Zenzeri L, Agostinelli S, De Feo P. Childhood obesity: prevention and strategies of intervention. A systematic review of school-based interventions in primary schools. J Endocrinol Invest [Internet]. 2014;37(12):1155–1164. Available from: https://link.springer.com/content/pdf/10.1007%2Fs40618-014-0153-y.pdf
17. Mikkelsen M V., Husby S, Skov LR, Perez-Cueto FJA. A systematic review of types of healthy eating interventions in preschools. Nutr J. 2014;13(1):1–19.
18. Saraf DS, Nongkynrih B, Pandav CS, Gupta SK, Shah B, Kapoor SK, et al. A systematic review of school-based interventions to prevent risk factors associated with noncommunicable diseases. Asia-Pacific J Public Heal. 2012;24(5):733–52.
19. Naylor PJ, Nettlefold L, Race D, Hoy C, Ashe MC, Wharf Higgins J, et al. Implementation of school based physical activity interventions: A systematic review. Prev Med (Baltim). 2015 Mar 1;72:95–115.
20. Van Cauwenberghe E, Maes L, Spittaels H, van Lenthe FJ, Brug J, Oppert JM, et al. Effectiveness of school-based interventions in Europe to promote healthy nutrition in children and adolescents:

systematic review of published and 'grey' literature. Br J Nutr [Internet]. 2010 Mar 14 [cited 2018 Oct 30];103(06):781. Available from: http://www.journals.cambridge.org/abstract_S0007114509993370

21. Singh AS, Mulder C, Twisk JWR, Van Mechelen W, Chinapaw MJM. Tracking of childhood overweight into adulthood: a systematic review of the literature. Obes Rev. 2008;9(5):474–88.
22. Schwartz AE, Rothbart MW. Let them eat lunch: The impact of universal free meals on student performance. Journal of Policy Analysis and Management. 2020 Mar;39(2):376-410.
23. Asigbee FM, Whitney SD, Peterson CE. The link between nutrition and physical activity in increasing academic achievement. Journal of School Health. 2018 Jun;88(6):407-15.
24. Willeboordse M, Jansen MW, van den Heijkant SN, Simons A, Winkens B, de Groot RHM, et al. The Healthy Primary School of the Future: study protocol of a quasi-experimental study. BMC Public Health [Internet]. 2016 Dec;16(1):639. Available from: http://bmcpublichealth.biomedcentral.com/articles/10.1186/s12889-016-3301-9
25. Bartelink NHM, van Assema P, Kremers SPJ, Savelberg HHCM, Oosterhoff M, Willeboordse M, et al. One- and two-year effects of the healthy primary school of the future on children's dietary and physical activity behaviours: A quasi-experimental study. Nutrients. 2019;11(3):1–18.
26. Willeboordse M, Bartelink NHM, Van Assema P, Kremers SPJ, Savelberg HHCM, Hahnraths MTH, et al. Battling the obesity epidemic with a school-based intervention: Long-term effects of a quasi-experimental study. Fürnsinn C, editor. PLoS One [Internet]. 2022 Sep 27 [cited 2022 Sep 27];17(9):e0272291. Available from: https://journals.plos.org/plosone/article?id=10.1371/journal.pone.0272291
27. Bartelink NHM, van Assema P, Kremers SPJ, Savelberg HHCM, Oosterhoff M, Willeboordse M, et al. Can the Healthy Primary School of the Future offer perspective in the on-going obesity epidemic in young children? – a quasi-experimental study. BMJ Open. 2019;9(e030676).
28. Centraal Instituut voor Toetsontwikkeling (CITO). *National Standardized Tests for Dutch Primary Education 2024* [Internet]. Arnhem: CITO; 2024 [cited 2024 Dec 3]. Available from: https://www.cito.nl
29. Gezondheidsraad. *Dutch Dietary Guidelines 2015*. The Hague: Gezondheidsraad; 2015.
30. Boudewijns EA, Pepels JJS, van Kann D, Konings K, van Schayck CP, Willeboordse M. Non-response and external validity in a school-based quasi-experimental study 'The Healthy Primary School of the Future': A cross-sectional assessment. Prev Med Reports [Internet]. 2019;14(100874). Available from:

https://doi.org/10.1016/j.pmedr.2019.100874

31. Vermeer A, Boot N, Hesdahl M, Janssen-Goffin M, Linssen E, Rutten N, et al. Lokale rapporten Volksgezondheid Toekomst Verkenning: Een nieuwe kijk op gezondheid in Heerlen, Kerkrade, Landgraaf en Brunssum; [Local reports on Public Health Development: A new perspective on health in Heerlen, Kerkrade, Landgraaf and Brunssum]. Geleen; 2014.
32. Cohen J. Statistical power analysis for the behavioral sciences. routledge; 2013 May 13.
33. Donnelly JE, Hillman CH, Castelli D, Etnier JL, Lee S, Tomporowski P, Lambourne K, Szabo-Reed AN. Physical activity, fitness, cognitive function, and academic achievement in children: a systematic review. Medicine and science in sports and exercise. 2016 Jun;48(6):1197.
34. Singh AS, Saliasi E, Van Den Berg V, Uijtdewilligen L, De Groot RH, Jolles J, Andersen LB, Bailey R, Chang YK, Diamond A, Ericsson I. Effects of physical activity interventions on cognitive and academic performance in children and adolescents: a novel combination of a systematic review and recommendations from an expert panel. British journal of sports medicine. 2019 May 1;53(10):640-7.
35. Muntaner-Mas A, Morales JS, Martínez-de-Quel Ó, Lubans DR, García-Hermoso A. Acute effect of physical activity on academic outcomes in school-aged youth: A systematic review and multivariate meta-analysis. Scandinavian Journal of Medicine & Science in Sports. 2024 Jan;34(1):e14479.
36. Burrows T, Goldman S, Olson RK, Byrne B, Coventry WL. Associations between selected dietary behaviours and academic achievement: A study of Australian school aged children. Appetite. 2017 Sep 1;116:372-80.
37. Florence MD, Asbridge M, Veugelers PJ. Diet quality and academic performance. Journal of school health. 2008 Apr;78(4):209-15.
38. Ritchie SJ, Bates TC. Enduring links from childhood mathematics and reading achievement to adult socioeconomic status. Psychological science. 2013 Jul;24(7):1301-8.
39. Hamilton L. *The role of the home literacy environment in the early literacy development of children at family-risk of dyslexia* (Doctoral dissertation, University of York).
40. Jeynes W. *Parental involvement and academic success* [Internet]. 1st ed. New York: Routledge; 2010 [cited 2024 Dec 3]. Available from: https://www.taylorfrancis.com/books/mono/10.4324/9780203843444/parental-involvement-academic-success-william-jeynes
41. Basch CE. Healthier students are better learners: A missing link in school reforms to close the

achievement gap. Journal of school health. 2011 Oct;81(10):593-8.

42. ParnasSys. *Leerling Administratie Systeem (LAS)* [Internet]. Topicus Onderwijs; [cited 2024 Dec 3]. Available from: https://www.parnassys.nl

Supplementary Materials

Appendix I. Observed mean test score (mathematics and reading comprehension) and SDs for each intervention group after one, two, three, and four years of exposure (delta1-delta4).

		**Full HPSF**		**Partial HPSF**		**Control**	
		**n**	**Mean (SD)**	**n**	**Mean (SD)**	**n**	**Mean (SD)**
**Mathematics**	**delta1**	212	147.2 (50.7)	139	103.2 (48.2)	342	129.2 (54.5)
	**delta2**	307	164.2 (52.3)	189	130.7 (50.8)	471	147.6 (55.2)
	**delta3**	481	170.4 (54.2)	285	147.5 (49.5)	733	158.0 (53.3)
	**delta4**	328	209.9 (42.1)	208	182.6 (54.1)	496	195.0 (47.7)
**Reading Comprehension**	**delta1**	213	30.2 (9.5)	135	25.7 (12.7)	305	28.1 (11.4)
	**delta2**	449	31.7 (10.3)	233	30.3 (9.9)	482	32.5 (11.8)
	**delta3**	404	36.6 (12.9)	237	33.8 (11.1)	630	35.3 (13.0)
	**delta4**	255	45.4 (12.3)	154	44.0 (12.5)	392	41.9 (13.4)

Abbreviations; HPSF = Healthy Primary School of the Future, SD = standard deviation.

Appendix II. Theoretical example of effect of full HPSF and control on mathematics

The following numbers were based on observed values from a child on a control school, estimated mean differences between full HPSF and control (Table 2), and national averages calculated from Pupil Administration System (LAS) ParnasSys database [42]

Control Group:

- Change in scores: 17.5 (Year 1), 8.5 (Year 2), 12.5 (Year 3), 13.2 (Year 4)
- Average Increase: (17.5+8.5+12.5+13.2)/4 = 12.9 points/year

Full HPSF Group:

- Change in scores: 23.7 (Year 1), 10.7 (Year 2), 12.0 (Year 3), 19.7 (Year 4)
- Average Increase: (23.7+10.7+12.0+19.7)/4 = 16.5 points/year

National Average:

- Change in scores: 19.1 (Year 1), 17.4 (Year 2), 12.1 (Year 3), 13.2 (Year 4)
- Average Increase: (19.1+17.4+12.1+13.2)/4 = 15.5 points/year

Figure 1a

Click here to access/download;Figure;Figure 1a.tif

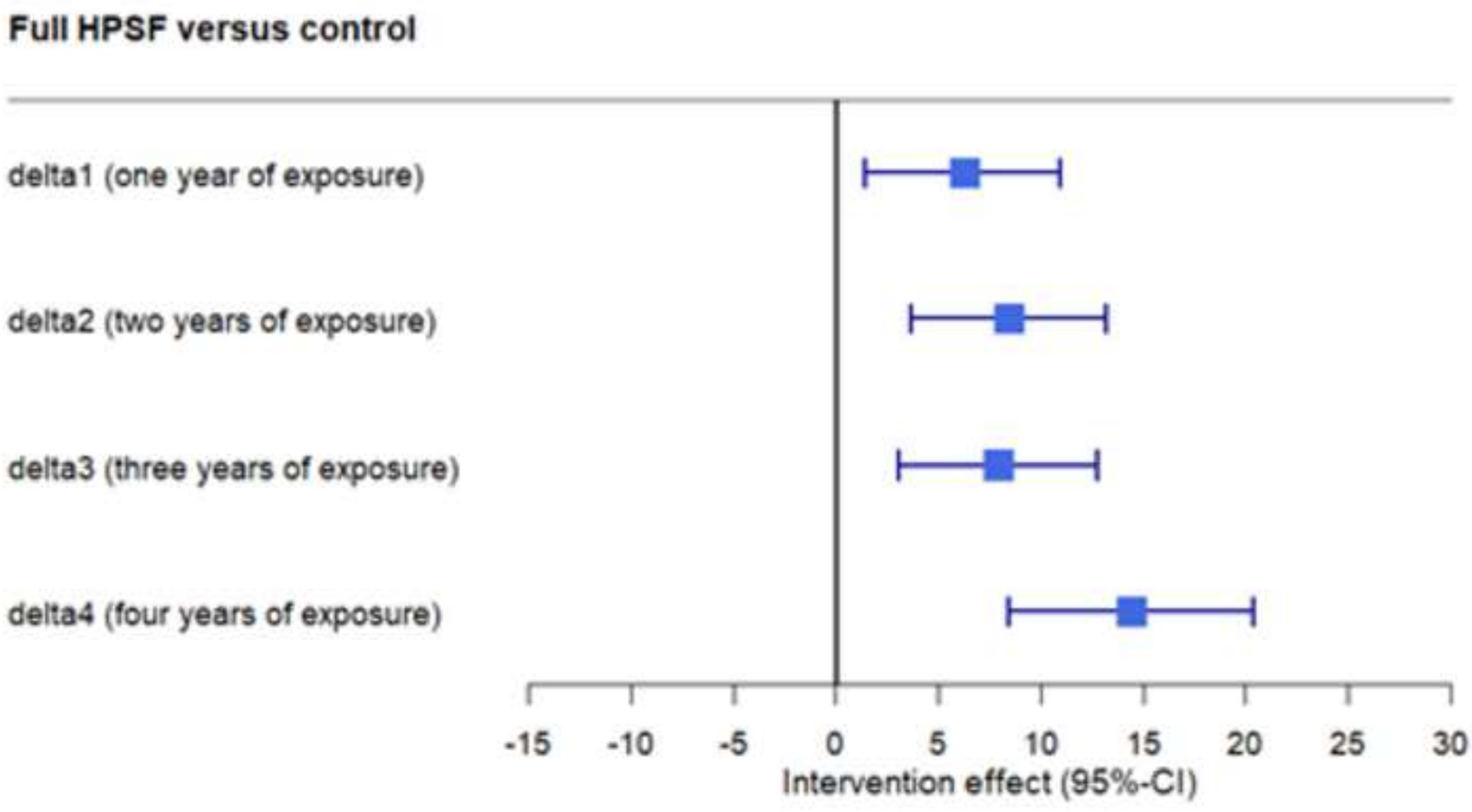

Figure 1b

Click here to access/download;Figure;Figure 1b.tif

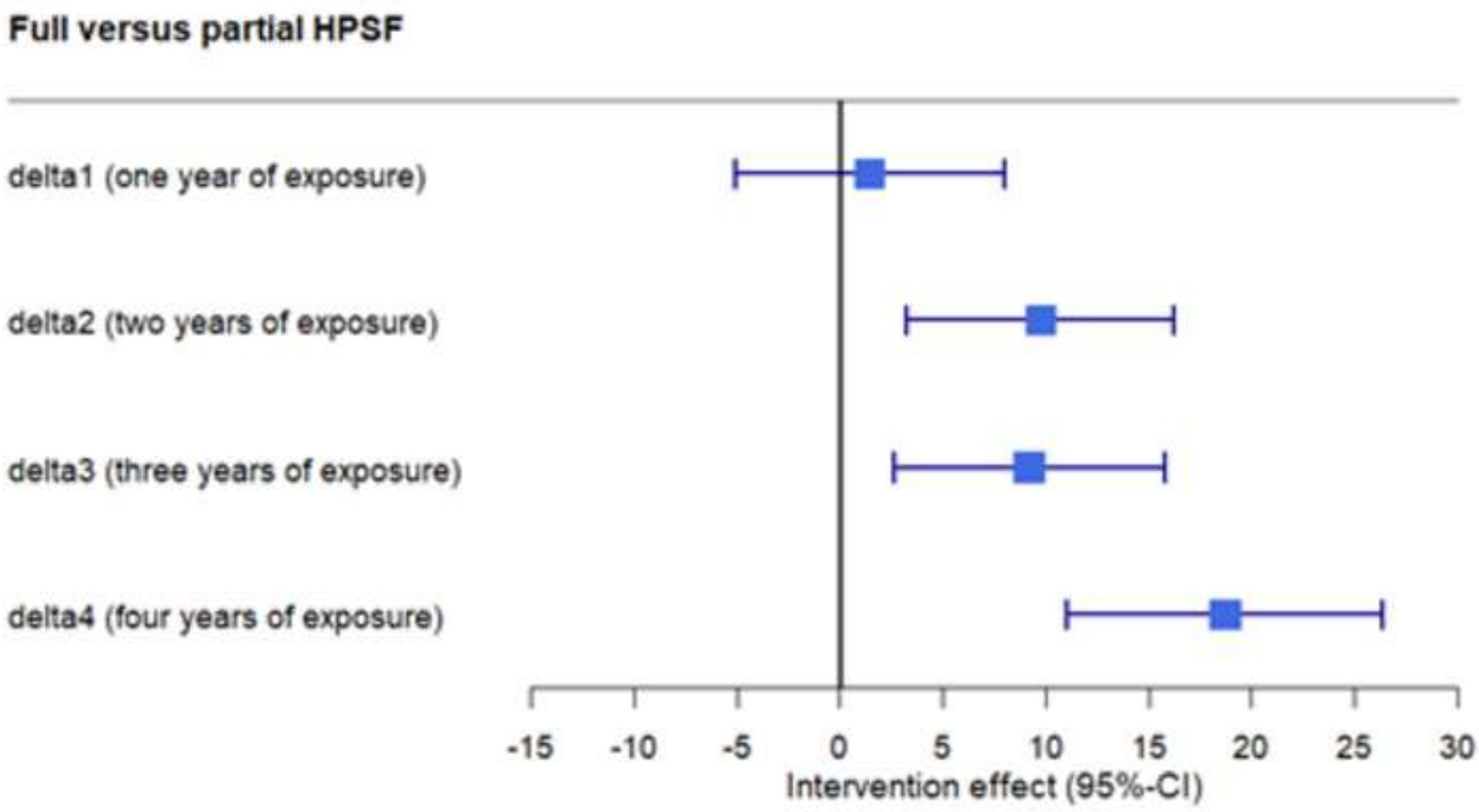

Figure 1c

Click here to access/download;Figure;Figure 1c.tif

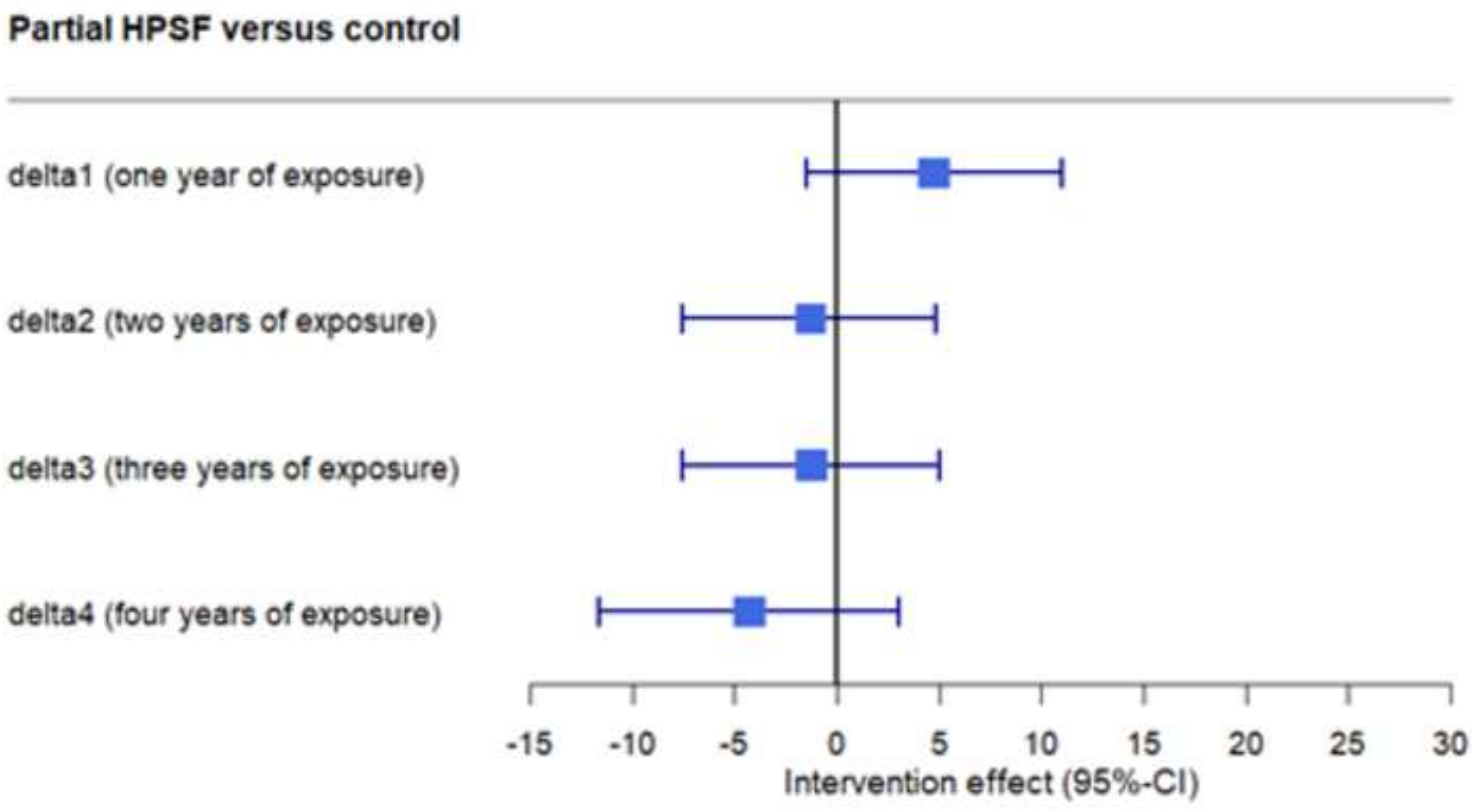

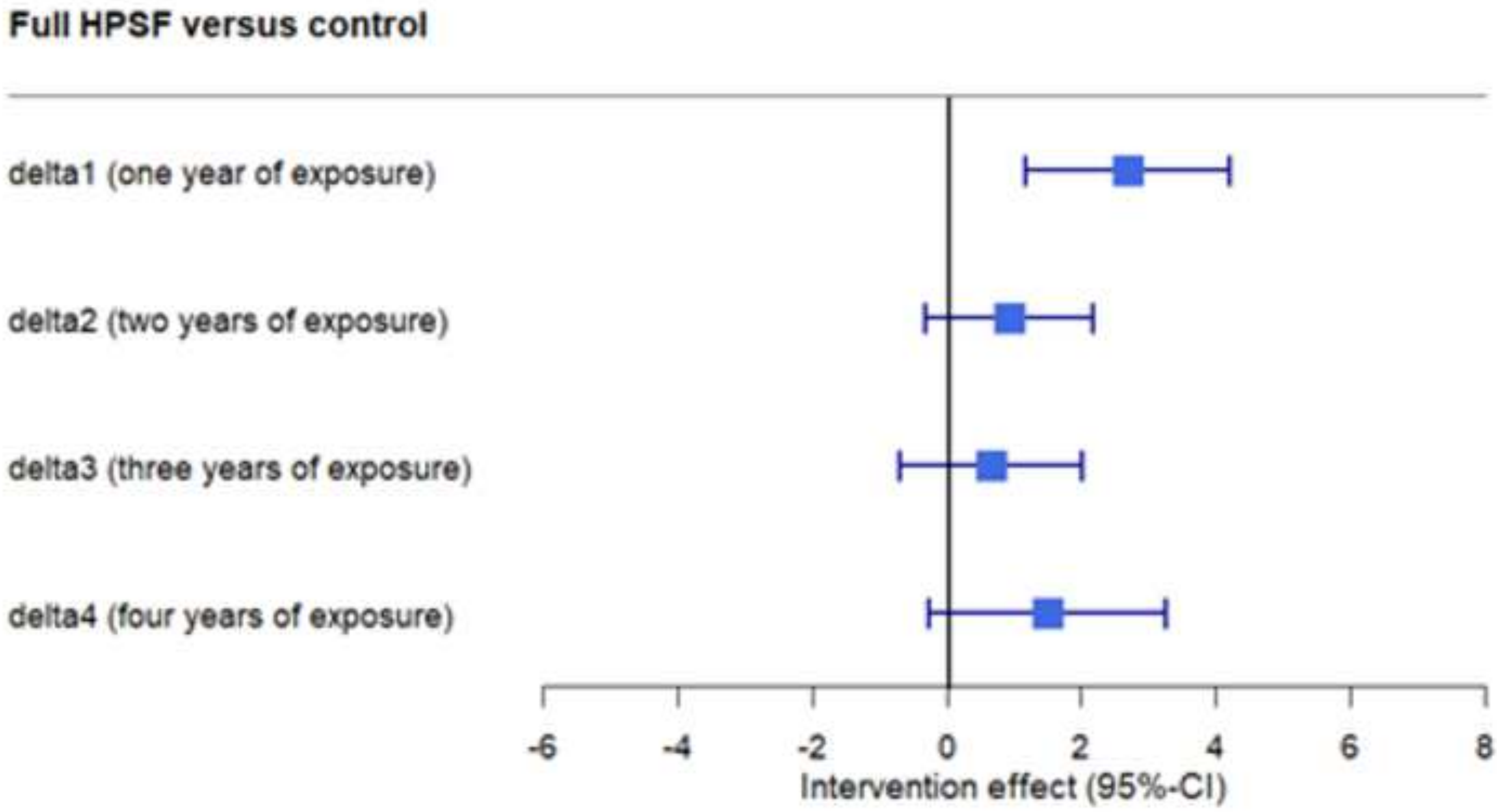
Full HPSF versus control
delta1 (one year of exposure)
delta2 (two years of exposure)
delta3 (three years of exposure)
delta4 (four years of exposure)
-6
-4
-2
0
2
4
6
8
Intervention effect (95%-CI)

Figure 2b

Click here to access/download;Figure;figure 2b.tif

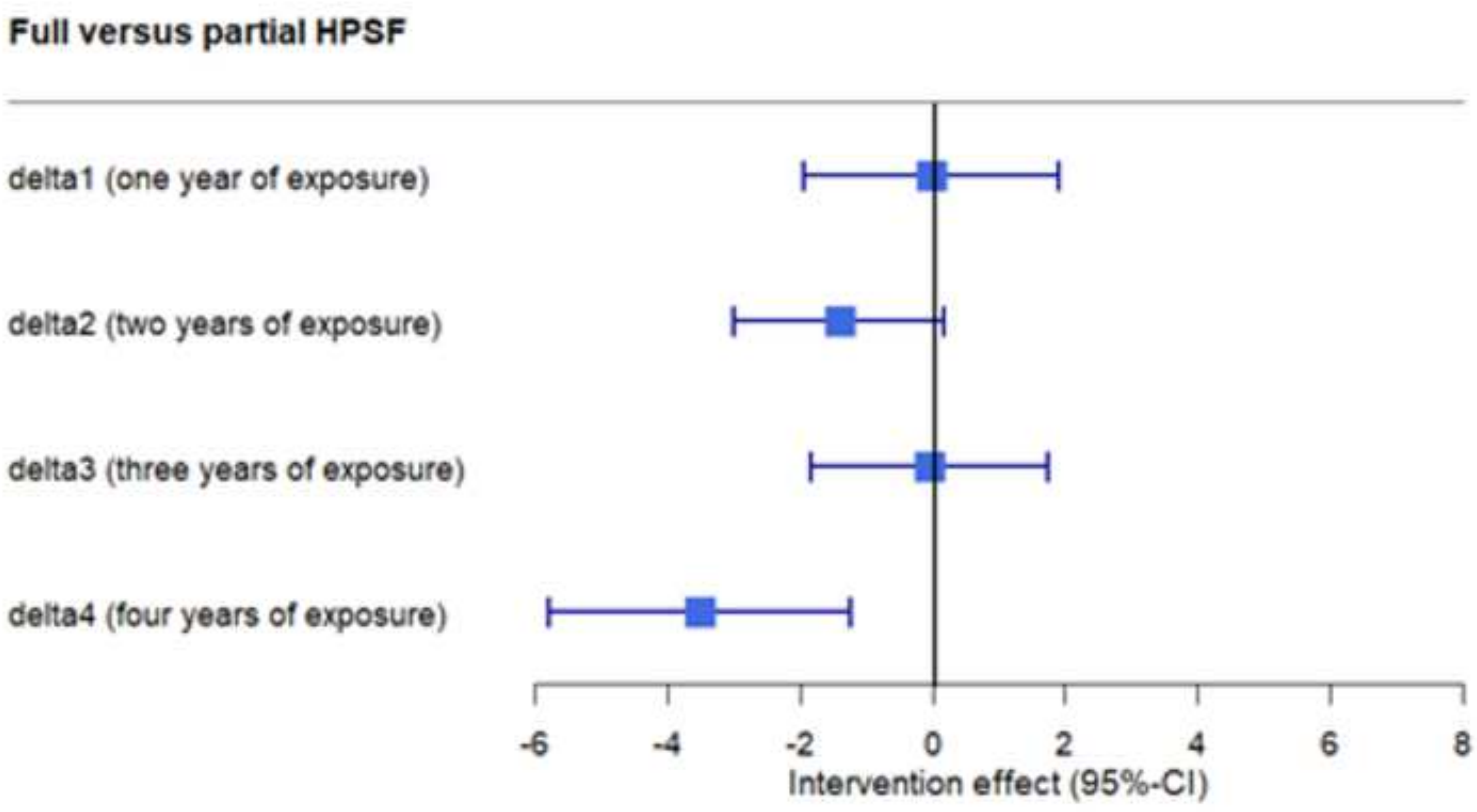

Figure 2c

Click here to access/download;Figure;figure 2c.tif

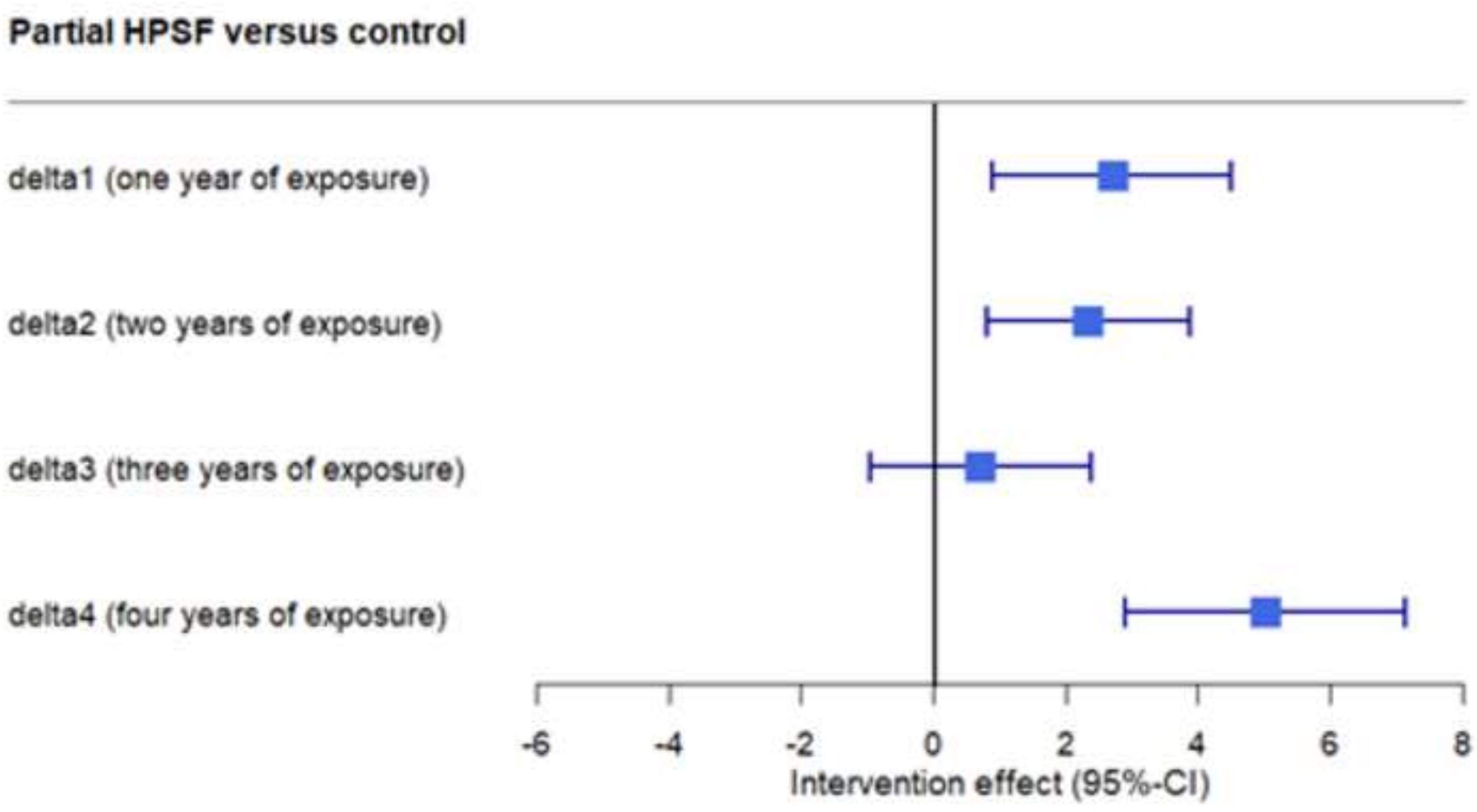

Supplement: Attachment — Submitted filename: editPONE-D-24-56254.pdf [file pone.0334638.s007.pdf]
