# Supplementary material for: Food for thought? The effects of the Healthy Primary School of the Future on children’s educational outcomes
Source: PLoS One. 2026 Jun 24;21(6):e0334638. doi: 10.1371/journal.pone.0334638 (PMC13293421; doi:10.1371/journal.pone.0334638)
Supplement: Attachment — Submitted filename: rebuttal letter.docx [file pone.0334638.s008.docx]

**Manuscript title:** *Food for Thought? The Effects of the Healthy Primary School of the Future on Children’s Educational Outcomes*

**Authors:** van Engelen BHW, Hahnraths MTH, Winkens B, Schils t, OCP van Schayck

We thank the reviewers and the academic editor for their constructive comments and careful reading of our manuscript. Below we provide a point-by-point response. Reviewer comments are shown in *italics*, and our replies follow each point. All suggested changes have been incorporated into the revised manuscript.

**Reviewer #1**

*This is an important study, with a clear message. Especially full HPSF intervention (both healthy lunches and structured PA sessions at school during 4 years) had a strong positive effect on children's (aged 6-12 years) mathematical performance in Netherlands (Parkstad region). This is especially important, because this region is known as a low to moderate socioeconomic area and as the authors state: Equity in education at primary school is an important issue in education, where it is aimed that all students are given equal opportunities to develop their talents, regardless of their SES, ethnicity, or other circumstances. Unfortunately the authors could not analyse the effect of SES-background on the findings, because of acceptable reasons.
The authors hope that the study would be replicated in other SES environments and in other countries, I agree with this.*

**Response:**
We thank the reviewer for the positive and supportive feedback. We agree that equity in education is a key issue and that replication in other socioeconomic contexts is needed. As noted, we could not include SES in the analyses. This was not due to poor documentation, but because SES information in Dutch primary schools is only available via parental questionnaires, which had a ~50% non-response rate. Non-response is known to be common among families with lower SES or non-Dutch language backgrounds (Boudewijns et al., 2019). This resulted in too many missing values to allow for reliable adjustment or multiple imputation. While multiple imputations are technically possible even with a high percentage of missing data (e.g., 50%), its reliability depends on having other variables that are strongly correlated with the variable containing the missing values. In this case, the outcome variable is likely related to socioeconomic status (SES), but not strongly enough to produce reliable imputations. We also emphasize that all schools were located in the same low-to-moderate SES region under one school board, which reduces but does not eliminate SES heterogeneity. We have clarified this in the revised *Strengths and Limitations* section by adding a paragraph (page 12, lines 330–341):

‘The reason why we were unable to adjust for children’s socioeconomic background was that information on parental education (as a proxy for SES) is not routinely collected in Dutch primary education and was therefore only available via parental questionnaires. Unfortunately, a substantial proportion of parents (~50%) did not complete these questionnaires. Non-response is known to be more common among families with a lower SES or a non-Dutch language background [30]. While residual confounding cannot be excluded, several considerations may reduce this concern: (1) all schools were located in the same low-to-moderate SES region under one school board, limiting SES heterogeneity; and (2) a previous study (specifically designed to investigate the external validity of the study with the present study population [30]) confirmed that participants were broadly representative of the region in terms of sex (48% boys vs. 50% regionally) and socioeconomic status, as indicated by maternal educational level (25% low, 45% middle, 30% high vs. 27%, 42%, 31% regionally), as well as health-related characteristics. Nevertheless, replication of this study in other socioeconomic contexts is necessary to establish the generalizability of our findings.’

We also emphasize that all schools were located in the same low-to-moderate SES region under one school board, which reduces but may not fully eliminate SES heterogeneity.

**Reviewer #2**

*This manuscript by van Engelen et al. describes a nutrition and physical activity intervention in Dutch schools. The primary outcome was an improvement in math and reading scores compared to control schools. They showed that, in schools that had both a free lunch and additional physical activity intervention, students had higher math scores throughout the four-year period and higher reading scores in the first year of intervention.
Overall, this tackles an important question in the field—does providing health-targeted interventions in school improve academic results?
There are some weaknesses here to address. First, it’s unclear how the schools were chosen to be part of the intervention, partial intervention or control groups. Second, the major confounders for academic performance were not adequately measured. For those two reasons, it’s difficult for the reader to decide if the improvements in math and reading scores are related to the intervention or driven by another unmeasured/unaccounted for variable, namely the many socioeconomic drivers of academic performance. Another challenge here is that the reader is not well-guided to understand the degree of improvement in the academic scores. The improvements are statistically significant, but what does a low-to-moderate effect size mean in the real world? How does this compare to other interventions? How does this compare to the effects of SES factors?
It is admirable that a nutrition and movement intervention was studied for its effect on academic performance. Data like this should be more available for educators, school administrators and governments. However, this may be more suited as a quality improvement publication, perhaps in an education-focused journal.*

**Response:**

We thank the reviewer for the thoughtful review and for recognizing the importance of our research question. We appreciate your positive feedback on our manuscript, which investigates the impact of a nutrition and physical activity intervention on academic performance in Dutch schools.

We are glad you found our study's aim of examining whether health-targeted interventions in schools can improve academic results to be an important contribution to the field. Your summary of our key findings—that schools with both a free lunch and additional physical activity intervention saw sustained improvements in math scores and initial improvements in reading scores—accurately reflects the core results of our work.

Your comments are highly valuable and will help us improve the final manuscript. We are grateful for your time and expertise in reviewing our work. Please find below our response to your concerns.

*1. It’s unclear how the schools were chosen to be part of the intervention, partial intervention or control groups.*

**Response:**
We thank the reviewer for this comment. We would like to clarify that our study employed a longitudinal quasi-experimental design rather than a randomized controlled trial (RCT). We have now explicitly stated this in the *Abstract* (line 26), *Methods* (line 123), and *Discussion* (line 318). An RCT was not feasible in this context. Randomization on individual level was not possible as it would have required randomizing children across different schools. In addition, successful implementation depended on the cooperation and commitment of school boards, principals, teachers, and parents. For these reasons, voluntary participation at the school level was the only realistic option, making a quasi-experimental design the most appropriate and feasible choice. This is explained in detail in our publication describing the protocol and study design of this study [24]. We have also added this explanation in the Discussion line 381-384 to justify our methodological approach.

‘While some may view school health programs primarily as quality improvement, we emphasize that the HPSF was evaluated in a longitudinal quasi-experimental design with repeated, standardized national testing over four years. This methodological rigor, combined with a large dataset and robust analyses, allows us to provide scientific evidence on the academic effects of a complex school-based health intervention.’

2. *The major confounders for academic performance were not adequately measured.*

**Response:**

We agree that SES and other potential confounders are important. We actively sought SES data, but in Dutch primary education this information is only available through parental questionnaires. Unfortunately, about 50% of parents did not respond, and non-response was more common among families with a lower SES or a non-Dutch language background (Boudewijns et al., 2019: as explained, this previous study specifically designed to investigate the external validity of the study with the present study population [30]). This limitation reflects parental non-response and resulted in too many missing values for meaningful inclusion in our analyses.

Nevertheless, several considerations reduce the risk of bias. First, all schools were located in the same low-to-moderate SES region under one school board, which limits heterogeneity between schools. Second, Boudewijns et al. (2019) showed that participants in the HPSF trial were broadly representative of the regional school population: 25% of mothers had a low education level, 45% a middle level, and 30% a high level, compared with 27%, 42%, and 31% regionally. In terms of sex distribution, the sample (48% boys) was also comparable to the regional population (50% boys). Finally, health-related characteristics of participating children were consistent with regional averages.

Taken together, these findings suggest that although individual-level SES data were incomplete, the study population is likely representative of the wider regional context, thereby reducing the risk of systematic bias. In line with Reviewer 1’s comment, we note that the inability to analyze SES effects stems from well-documented reasons. Reviewer 1 also acknowledged this point and agreed that replication of the study in other SES environments and in other countries is important, and we explicitly highlight this recommendation in the revised manuscript. We have clarified and expanded this limitation in the revised manuscript by adding a paragraph to the discussion section (page 12, lines 330–341):

‘The reason why we were unable to adjust for children’s socioeconomic background was that information on parental education (as a proxy for SES) is not routinely collected in Dutch primary education and was therefore only available via parental questionnaires. Unfortunately, a substantial proportion of parents (~50%) did not complete these questionnaires. Non-response was more common among families with a lower SES or a non-Dutch language background [30]. This resulted in too many missing values to allow for meaningful inclusion of SES in the analyses. While residual confounding cannot be excluded, several considerations may reduce this concern: (1) all schools were located in the same low-to-moderate SES region under one school board, limiting SES heterogeneity; and (2) a previous study (specifically designed to investigate the external validity of the study with the present study population [30]) confirmed that participants were broadly representative of the region in terms of sex (48% boys vs. 50% regionally) and socioeconomic status, as indicated by maternal educational level (25% low, 45% middle, 30% high vs. 27%, 42%, 31% regionally), as well as health-related characteristics. Nevertheless, replication of this study in other socioeconomic contexts is necessary to establish the generalizability of our findings.’

3. *The reader is not well-guided to understand the degree of improvement in the academic scores. The improvements are statistically significant, but what does a low-to-moderate effect size mean in the real world? How does this compare to other interventions? How does this compare to the effects of SES factors?*

**Response:**
We appreciate this important comment. To facilitate interpretation, we presented all results in standardized effect sizes (Cohen’s d). In the revised manuscript, we have expanded the interpretation by providing a theoretical example:

- A child in a control school gained on average 12.9 mathematics points per year, compared to 16.5 points in a full HPSF school.
- The national average was 15.5 points per year.
- Thus, children in HPSF schools progressed faster than the national trend, while those in control schools fell behind.

To further illustrate this, we have added a new figure to Appendix III, showing the mathematics performance change after one year in control schools, full HPSF schools, and the national average. Although the standardized effect sizes were small-to-moderate in statistical terms, such differences may accumulate meaningful educational consequences. Direct comparison with other health-based interventions is unfortunately not possible, as no comparable studies have been published with standardized test outcomes. Likewise, we cannot directly compare SES effects in this dataset due to missing data. These clarifications have been added to the revised Discussion on page 13 line 350-359:

‘Interpretation of effect sizes in educational outcomes was challenging. We therefore presented all results also in standardized form (Cohen’s d) to facilitate comparison. To further contextualize the findings, we illustrated the effects using a theoretical example based on observed data: Children in control schools gained on average 12.9 mathematics points per year, compared to 16.5 points in full HPSF schools per year, while the national average was 15.5 points per year. Thus, children in HPSF schools progressed faster than the national trend, whereas those in control schools fell behind (Appendix III). Although the standardized effect sizes were small-to-moderate in statistical terms, such differences may accumulate over years to have meaningful educational consequences. Direct comparison with other health-based interventions is unfortunately not possible, as to our knowledge no comparable studies with standardized test outcomes have been published.

4. *This may be more suited as a quality improvement publication, perhaps in an education-focused journal.*

**Response:**

We respectfully disagree. The Healthy Primary School of the Future intervention was evaluated using a longitudinal quasi-experimental design with repeated national standardized tests over four years, involving rigorous statistical analysis. This goes beyond educational quality improvement reporting and provides scientific evidence on the academic impact of a complex school-based health intervention. Importantly, a previous study reporting on the long-term health effects of HPSF by Willeboordse et al. (2022) was earlier published in *PLOS ONE*, demonstrating that this journal considers school-based interventions with rigorous quasi-experimental designs suitable for its scope. According to the *PLOS ONE* mission statement, the journal welcomes “primary research from any discipline within science and medicine” that is methodologically rigorous and ethically conducted, regardless of perceived impact. We believe that our study clearly meets these criteria, as it applies to a robust design to address a relevant question at the intersection of public health, education, and social equity.

Furthermore, to facilitate a more generic interpretation of our outcomes, we have added Appendix III, which illustrates the practical meaning of effect sizes in terms of annual mathematics score gains compared to national averages. We have also strengthened the *Implications* section of the manuscript to emphasize the broader significance of these findings by adding the following to page 14 line 379-382:

‘While some may view school health programs primarily as quality improvement, we emphasize that the HPSF was evaluated in a longitudinal quasi-experimental design with repeated, standardized national testing over four years. This methodological rigor, combined with a large dataset and robust analyses, allows us to provide scientific evidence on the academic effects of a complex school-based health intervention.’

**Summary of Key Revisions**

1. Clarified recruitment and assignment of schools (Methods).
2. Expanded discussion of SES limitations, clarifying that missingness was due to parental non-response and not to poor documentation (Discussion 4.3).
3. Added contextualized interpretation of educational effect sizes with a practical example comparing control, intervention, and national averages (Discussion 4.2/4.3).
4. Strengthened justification that the study is hypothesis-driven research rather than education quality improvement (Discussion 4.4).

**Academic editor**

*1. Please ensure that your manuscript meets PLOS ONE's style requirements, including those for file naming. The PLOS ONE style templates can be found at*

[*https://journals.plos.org/plosone/s/file?id=wjVg/PLOSOne_formatting_sample_main_body.pdf*](https://journals.plos.org/plosone/s/file?id=wjVg/PLOSOne_formatting_sample_main_body.pdf) *and* 
[*https://journals.plos.org/plosone/s/file?id=ba62/PLOSOne_formatting_sample_title_authors_affiliations.pdf*](https://journals.plos.org/plosone/s/file?id=ba62/PLOSOne_formatting_sample_title_authors_affiliations.pdf)

**Response:**

We have carefully reviewed the PLOS ONE style requirements and updated our manuscript and file naming to ensure full compliance with the journal’s guidelines, following the provided templates.

*2. Thank you for stating the following financial disclosure:*

*“De OnderwijsMonitor Limburg was funded by Kennisas project Educatieve Agenda Limburg, Provincial Government of Limburg, Maastricht University, and primary and secondary schools in the province of Limburg. The HPSF project was funded by the Limburg provincial authorities, Project Number 200130003, by Friesland Campina, Project Number LLMV00, and by Maastricht University, Project Number 200130003.”*

*Please state what role the funders took in the study.  If the funders had no role, please state: "The funders had no role in study design, data collection and analysis, decision to publish, or preparation of the manuscript." If this statement is not correct you must amend it as needed.*

*Please include this amended Role of Funder statement in your cover letter; we will change the online submission form on your behalf.*

**Response:**

We thank the editor for pointing this out. We confirm that the funders had no role in study design, data collection and analysis, decision to publish, or preparation of the manuscript. We have added the following statement to our cover letter, as requested:

*“**The funders had no role in study design, data collection and analysis, decision to publish, or preparation of the manuscript.”*

*3. Please note that funding information should not appear in the Acknowledgments section or other areas of your manuscript. We will only publish funding information present in the Funding Statement section of the online submission form. Please remove any funding-related text from the manuscript.*

**Response:**

We have removed all funding-related text from the Acknowledgments and other sections of the manuscript, ensuring that funding information only appears in the Funding Statement section of the online submission form, in line with PLOS ONE requirements.

*4. We note that your Data Availability Statement is currently as follows:*

*“All relevant data are within the manuscript and its Supporting Information files.”*

*Please confirm at this time whether or not your submission contains all raw data required to replicate the results of your study. Authors must share the “minimal data set” for their submission. PLOS defines the minimal data set to consist of the data required to replicate all study findings reported in the article, as well as related metadata and methods (*[*https://journals.plos.org/plosone/s/data-availability#loc-minimal-data-set-definition*](https://journals.plos.org/plosone/s/data-availability#loc-minimal-data-set-definition)*).*

*For example, authors should submit the following data:*

*- The values behind the means, standard deviations and other measures reported;
- The values used to build graphs;
- The points extracted from images for analysis.*

*Authors do not need to submit their entire data set if only a portion of the data was used in the reported study.*

*If your submission does not contain these data, please either upload them as Supporting Information files or deposit them to a stable, public repository and provide us with the relevant URLs, DOIs, or accession numbers. For a list of recommended repositories, please see* [*https://journals.plos.org/plosone/s/recommended-repositories*](https://journals.plos.org/plosone/s/recommended-repositories)*.
If there are ethical or legal restrictions on sharing a de-identified data set, please explain them in detail (e.g., data contain potentially sensitive information, data are owned by a third-party organization, etc.) and who has imposed them (e.g., an ethics committee). Please also provide contact information for a data access committee, ethics committee, or other institutional body to which data requests may be sent. If data are owned by a third party, please indicate how others may request data access.*

**Response:**

We thank the reviewer and the editors for pointing out the requirements regarding data availability. We confirm that we have uploaded the minimal dataset required to replicate all study findings reported in our manuscript.

Specifically, we have now provided two Supporting Information files that contain the raw data underlying our analyses:

- 20241111_Mathematics_dataset_gender_age_SES
- 20241111_ReadingComprehension_dataset_gender_age_SES

These files include all variables necessary to replicate the reported results (including the values behind the descriptive statistics, inferential analyses, and figures). The datasets have been fully de-identified and are in line with ethical guidelines.

We have updated our Data Availability Statement accordingly to reflect that these files are provided as Supporting Information.

*5. PLOS requires an ORCID iD for the corresponding author in Editorial Manager on papers submitted after December 6th, 2016. Please ensure that you have an ORCID iD and that it is validated in Editorial Manager. To do this, go to ‘Update my Information’ (in the upper left-hand corner of the main menu), and click on the Fetch/Validate link next to the ORCID field. This will take you to the ORCID site and allow you to create a new iD or authenticate a pre-existing iD in Editorial Manager.*

**Response:**

Thank you for this reminder. The corresponding author has now registered an ORCID iD and validated it in Editorial Manager as requested.

*6. Your ethics statement should only appear in the Methods section of your manuscript. If your ethics statement is written in any section besides the Methods, please delete it from any other section.*

**Response:**

Thank you for your comment. We have revised the manuscript so that the ethics statement now appears only in the Methods section on page 5 lines 147-149, in accordance with the guidelines.

*7. We notice that your supplementary materials are included in the manuscript file. Please remove them and upload them with the file type 'Supporting Information'. Please ensure that each Supporting Information file has a legend listed in the manuscript after the references list.*

**Response:**

Thank you for pointing this out. We have removed the supplementary materials from the main manuscript file and uploaded them separately under the file type *Supporting Information*. We have also provided appropriate legends for each Supporting Information file, which are now listed in the manuscript after the references section, as requested.

*8. If the reviewer comments include a recommendation to cite specific previously published works, please review and evaluate these publications to determine whether they are relevant and should be cited. There is no requirement to cite these works unless the editor has indicated otherwise.*

**Response:**

We carefully reviewed the suggested publications and evaluated their relevance to our work. Based on this assessment, we have included the following reference in the revised manuscript (page 19-20, line 558-561):

42. Lobo E, Mukherjee D, Choudhury PK, Babu GR, Srinivas PN, van Schayck OCP. *Home-learning environment and cognitive and academic outcomes among children aged 4–8 years: A cross-sectional study from South India* [Internet]. *Dialog.* 2025 [cited 2025 Sep 9];100238. Available from: <https://doi.org/10.1016/j.dialog.2025.100238>

We thank both reviewers and the academic editor for their constructive input, which has significantly improved the clarity and rigor of the manuscript.
