# Supplementary material for: Food for thought? The effects of the Healthy Primary School of the Future on children’s educational outcomes
Source: PLoS One. 2026 Jun 24;21(6):e0334638. doi: 10.1371/journal.pone.0334638 (PMC13293421; doi:10.1371/journal.pone.0334638)
Supplement: Attachment — Submitted filename: Response to Reviewers 3 and 4.docx [file pone.0334638.s009.docx]

**Response to Reviewers 3 and 4**

We thank Reviewers 3 and 4 for their careful reading of the manuscript and their constructive feedback. We also thank the editor for the opportunity to revise the manuscript. Below, we address each comment point-by-point. Revisions have been made in close consultation with the statistical expert on the author team (Professor Bjorn Winkens) and are reflected in the revised manuscript.

**1. Study design clarification**

**Reviewer comment:**
The study more closely resembles a natural experiment / observational longitudinal design rather than a classical quasi-experimental study.

**Response:**
We thank the reviewer for this important comment and appreciate the opportunity to further clarify the study design. While schools indeed participated on a voluntary basis and allocation was not randomized, the study was intentionally designed and implemented as a quasi-experimental study, consistent with the original HPSF study protocol (Willeboordse et al., 2016). The intervention conditions (full HPSF, partial HPSF, control), timing of implementation, predefined outcomes, and longitudinal follow-up were specified a priori and systematically evaluated, which aligns with established definitions of quasi-experimental designs. Moreover, the full result paper presenting all obesity-related outcomes of the intervention in this quasi-experimental study was earlier published in PlosOne (Willeboordse et al, 2022) and was presented as such. No other reviewers in this as well as the preceding peer-review by PlosOne have ever suggested to relabel our study as a natural experiment or observational study.

At the same time, we agree that the absence of random allocation limits causal inference. To address this, we have clarified in the Discussion that, although the study follows a quasi-experimental framework, results should be interpreted with appropriate caution regarding causality.

**Suggested manuscript text (Limitations page 13 lines 337-345)**

Implementation of the core components of the HPSF, including the healthy lunch and structured physical activity, was monitored through direct and intensive involvement of the research team, with regular contact at both the school and school board levels to assess whether implementation aligned with the agreed implementation plan. This multi-level approach was intended to address a well-known challenge in educational research, namely that interventions are frequently not implemented as intended. Engagement at both the school and governance levels supported internal ownership of the intervention and facilitated sustained implementation over time. Moreover, consistent with the conceptualization of the HPSF as a complex adaptive system, the accompanying process evaluation emphasized not only fidelity but also context-specific adaptations, as strict fidelity does not necessarily translate into greater effectiveness in complex educational settings

**2. Confounding variables and selection bias**

**Reviewer comment:**
The absence of key covariates such as SES and the home learning environment raises concerns about selection bias and limits causal inference.

**Response:**
We agree with the reviewer. Unfortunately, the substantial amount of missing data on SES and home learning environment variables precluded their inclusion in the final models. While this may indeed introduce residual confounding and selection bias, we included all relevant variables that could be reliably analyzed within the scope of the present study.

**Revision made:**
We have strengthened the limitations section by explicitly discussing the potential impact of unmeasured confounding and selection bias, and by tempering causal language throughout the manuscript.

**Suggested manuscript text (Limitations page 14 lines 375-383)**

“An important limitation is that key potential confounders, such as socioeconomic status, the home learning environment and comprehension reading at baseline, could not be included due to substantial missing data. As a result, residual confounding and selection bias cannot be ruled out. Schools opting into the HPSF may differ systematically from control schools in unobserved characteristics related to both health promotion and academic outcomes, which limits causal interpretation of the findings.

Previous research on the HPSF/GBT cohort indicates that although evidence for a healthy-volunteer effect is limited, responders were more likely to have higher educated parents and differed from the national population on several sociodemographic characteristics. Consequently, external validity appears to be high with respect to the regional population but more limited when comparisons are made at the national level.(30)’’

**3. Reading comprehension outcomes**

**Reviewer comment:**
Results for reading comprehension are inconsistent across years, and the lack of baseline data complicates interpretation. Further discussion of why partial HPSF schools outperform in some years is recommended.

**Response:**

The inconsistency in the results across years indeed complicates the interpretation. We have therefore expanded the Discussion to more explicitly address this variability and to propose plausible explanations for the relatively better performance of partial HPSF schools in certain years.

**Suggested manuscript text (Limitations page 12 rules 315-319):**

As for the larger effect of partial HPSF over full HPSF, this difference was only statistically significant at year 4 and showed no consistent pattern (almost no difference at year 1 and year 3). This difference over time might also be partially explained by a possible difference between the partial and full HPSF schools at baseline, which could not be accounted for due to the number of missing values. This is also mentioned as one of the main limitations below.

**4. Intervention fidelity**

**Reviewer comment:**
More information on the consistency of implementing the healthy lunch and physical activity components over the four-year period would improve interpretability.

**Response:**

We thank the reviewer for this important observation. Indeed, many educational interventions are not implemented as originally intended, which can substantially affect interpretability of findings. In the present study, implementation of the core components (healthy lunch and structured physical activity) was therefore closely monitored through direct and intensive involvement of the research team, including regular contact with schools to assess adherence to the implementation plan. In addition, monitoring occurred not only at the school level but also via close collaboration with school boards, thereby embedding the intervention within existing governance structures and enhancing internal ownership. This multi-level engagement has now been more clearly described in the Discussion section.

**Suggested manuscript text (Limitations page 13 lines 337-345)**

Implementation of the core components of the HPSF, including the healthy lunch and structured physical activity, was monitored through direct and intensive involvement of the research team, with regular contact at both the school and school board levels to assess whether implementation aligned with the agreed implementation plan. This multi-level approach was intended to address a well-known challenge in educational research, namely that interventions are frequently not implemented as intended. Engagement at both the school and governance levels supported internal ownership of the intervention and facilitated sustained implementation over time. Moreover, consistent with the conceptualization of the HPSF as a complex adaptive system, the accompanying process evaluation emphasized not only fidelity but also context-specific adaptations, as strict fidelity does not necessarily translate into greater effectiveness in complex educational settings

**Reviewer #4**

We thank Reviewer #4 for the detailed and technically oriented comments, which substantially improved the clarity and rigor of the manuscript.

**1. Line-by-line statistical clarifications (Lines 123–217)**

**Comment (Lines 123, 145–146):**
The sentence requires revision.

**Response:**
The indicated sentences have been revised to improve clarity and readability. If further revisions are needed, we are more than willing to accommodate this.

**Comment (Lines 188–189):**
The sentence could be improved, e.g. *Proportions were compared using Pearson’s chi-square tests, and mean values were compared using one-way ANOVA.*

**Response:**
The text has been revised accordingly, and the statistical tests are now explicitly stated.

**Comment (Lines 189–190):**
The sentence requires revision, e.g. *If the homoscedasticity assumption (assessed using Levene’s test) was violated, Welch’s test was used for overall comparisons, and the Games–Howell test was used for pairwise comparisons.*

**Response:**
The text has been revised accordingly to clearly describe the assessment of homoscedasticity and the use of Welch’s and Games–Howell tests when this assumption was violated.

**Comment (Lines 193–197):**
The statistical test is to be stated. If GEE is employed, the purpose should be clearly highlighted (e.g., to estimate the average intervention effect across all participants).

**Response:**
The statistical approach has been clarified. The exact model used is now explicitly stated, and its purpose—estimating average intervention effects over time—has been clearly described.

**Comment (Lines 202–204):**
The sentence requires revision. The exact name of the statistical test (e.g. LMM, MMRM) should be mentioned, and the sentence should be tailored to the specific test employed.

**Response:**
This section has been revised accordingly. The analysis is now explicitly described as a *mixed model for repeated measures (MMRM)*, and the description of the model and estimation approach has been clarified.

**Comment (Lines 204–205):**
For the corrected mean difference, state that it is adjusted for variables such as…

**Response:**
The manuscript now explicitly states which covariates were included in the adjustment of corrected mean differences (i.e. age, sex, test version, and baseline score for mathematics).

**Comment (Line 209):**
The sentence could be improved, e.g. *To adjust for multiple group comparisons, the Bonferroni method was applied.*

**Response:**
The sentence has been revised to:
*“To adjust for multiple group comparisons, the Bonferroni method was applied (significance level α = 0.05/3 = 0.0167).”*

**Comment (Lines 214, 217, etc.):**
“Was made” should be rephrased as “was conducted”.

**Response:**
This has been revised throughout the manuscript.

**Comment (Line 227):**
The time point should be stated. Alignment of the figures requires cosmetic changes.

**Response:**

We thank the reviewer for this comment. However, it was not entirely clear to us which specific time point or figure alignment issue this comment refers to. We have therefore carefully checked the manuscript and figures and confirmed that all time points are clearly indicated and that the figures are consistently aligned. We would be happy to address this further should the reviewer be able to clarify the comment.

**Comment (Lines 255, 262, 547):**
The Δ1–Δ4 notation may be confusing. It is suggested to highlight this as Δ1, Δ2, Δ3, Δ4.

**Response:**
This notation has been clarified throughout the text, tables, and figure captions.

**Comment:**
In the statistical analyses section of the Methods, ensure that all statistical tests mentioned are clearly indicated in the Results, and include a statement confirming that assumptions were assessed and fulfilled.

**Response:**
Consistency between the Methods and Results sections has been ensured. All statistical tests described in the Methods are explicitly reported in the Results (e.g. Pearson’s chi-square test, Welch’s test, Games–Howell test). In addition, the following statements were added:

- **Methods (blz en regels toevoegen):**
  *“The normality assumption of the residuals was checked per group and per timepoint (exposure) using histograms and Q–Q plots.”*
- **Results (blz en regels toevoegen:**
  *“All intervention effects were assessed using mixed models for repeated measures (MMRM), and all model assumptions were met.”*

**Comment:**
For any statistical tests or analyses described in the Methods, any important details or main output not presented in the main manuscript should be provided as supplementary files.

**Response:**
Important details were added to the manuscript (Pearson chi-square value, Welch F-value) as well as to a supplementary table (Mixed model for repeated measures results, including the overall F-test values and p-values for the interaction effect between group and exposure as well as the variance-covariance matrix of the repeated measures).

**Comment:**
A flow chart should be included, if possible, to illustrate how participants were selected and allocated to the intervention groups.

**Response:**
A flow chart illustrating participant selection and allocation to intervention groups has been added to the Supplementary Materials.

**Comment:**
The numbering for each title/subtitle should be omitted.

**Response:**
All numbering of titles and subtitles has been removed in accordance with journal formatting requirements.

**Closing statement**

We believe these revisions substantially improve methodological transparency, statistical clarity, and interpretability of the findings. We thank Reviewers 3 and 4 for their constructive feedback, which has strengthened the manuscript.
