# Supplementary material for: Food for thought? The effects of the Healthy Primary School of the Future on children’s educational outcomes
Source: PLoS One. 2026 Jun 24;21(6):e0334638. doi: 10.1371/journal.pone.0334638 (PMC13293421; doi:10.1371/journal.pone.0334638)
Supplement: Peer review history

# Peer review: Decision Letter 0

**DOI:** 10.1371/journal.pone.0334638.r001

**Authors:** Stijn Michielse

20 Aug 2025

PONE-D-24-56254

Food for Thought? The Effects of the Healthy Primary School of the Future on Children’s Educational Outcomes

PLOS ONE

Dear Dr. van Engelen,

Thank you for submitting your manuscript to PLOS ONE. After careful consideration, we feel that it has merit but does not fully meet PLOS ONE’s publication criteria as it currently stands. Therefore, we invite you to submit a revised version of the manuscript that addresses the points raised during the review process.

Please submit your revised manuscript by Oct 04 2025 11:59PM. If you will need more time than this to complete your revisions, please reply to this message or contact the journal office at plosone@plos.org. When you're ready to submit your revision, log on to https://www.editorialmanager.com/pone/ and select the 'Submissions Needing Revision' folder to locate your manuscript file.

Please include the following items when submitting your revised manuscript:

- A rebuttal letter that responds to each point raised by the academic editor and reviewer(s). You should upload this letter as a separate file labeled 'Response to Reviewers'.
- A marked-up copy of your manuscript that highlights changes made to the original version. You should upload this as a separate file labeled 'Revised Manuscript with Track Changes'.
- An unmarked version of your revised paper without tracked changes. You should upload this as a separate file labeled 'Manuscript'.

If you would like to make changes to your financial disclosure, please include your updated statement in your cover letter. Guidelines for resubmitting your figure files are available below the reviewer comments at the end of this letter.

If applicable, we recommend that you deposit your laboratory protocols in protocols.io to enhance the reproducibility of your results. Protocols.io assigns your protocol its own identifier (DOI) so that it can be cited independently in the future. For instructions see: https://journals.plos.org/plosone/s/submission-guidelines#loc-laboratory-protocols. Additionally, PLOS ONE offers an option for publishing peer-reviewed Lab Protocol articles, which describe protocols hosted on protocols.io. Read more information on sharing protocols at https://plos.org/protocols?utm_medium=editorial-email&utm_source=authorletters&utm_campaign=protocols.

We look forward to receiving your revised manuscript.

Kind regards,

Stijn Michielse, PhD

Academic Editor

PLOS ONE

Journal Requirements:

When submitting your revision, we need you to address these additional requirements.

1. Please ensure that your manuscript meets PLOS ONE's style requirements, including those for file naming. The PLOS ONE style templates can be found at

https://journals.plos.org/plosone/s/file?id=wjVg/PLOSOne_formatting_sample_main_body.pdf and

https://journals.plos.org/plosone/s/file?id=ba62/PLOSOne_formatting_sample_title_authors_affiliations.pdf

2. Thank you for stating the following financial disclosure:

“De OnderwijsMonitor Limburg was funded by Kennisas project Educatieve Agenda Limburg, Provincial Government of Limburg, Maastricht University, and primary and secondary schools in the province of Limburg. The HPSF project was funded by the Limburg provincial authorities, Project Number 200130003, by Friesland Campina, Project Number LLMV00, and by Maastricht University, Project Number 200130003.”

Please state what role the funders took in the study.  If the funders had no role, please state: "The funders had no role in study design, data collection and analysis, decision to publish, or preparation of the manuscript." If this statement is not correct you must amend it as needed.

Please include this amended Role of Funder statement in your cover letter; we will change the online submission form on your behalf.

3. Please note that funding information should not appear in the Acknowledgments section or other areas of your manuscript. We will only publish funding information present in the Funding Statement section of the online submission form. Please remove any funding-related text from the manuscript.

4. We note that your Data Availability Statement is currently as follows:

“All relevant data are within the manuscript and its Supporting Information files.”

Please confirm at this time whether or not your submission contains all raw data required to replicate the results of your study. Authors must share the “minimal data set” for their submission. PLOS defines the minimal data set to consist of the data required to replicate all study findings reported in the article, as well as related metadata and methods (https://journals.plos.org/plosone/s/data-availability#loc-minimal-data-set-definition).

For example, authors should submit the following data:

- The values behind the means, standard deviations and other measures reported;

- The values used to build graphs;

- The points extracted from images for analysis.

Authors do not need to submit their entire data set if only a portion of the data was used in the reported study.

If your submission does not contain these data, please either upload them as Supporting Information files or deposit them to a stable, public repository and provide us with the relevant URLs, DOIs, or accession numbers. For a list of recommended repositories, please see https://journals.plos.org/plosone/s/recommended-repositories.

If there are ethical or legal restrictions on sharing a de-identified data set, please explain them in detail (e.g., data contain potentially sensitive information, data are owned by a third-party organization, etc.) and who has imposed them (e.g., an ethics committee). Please also provide contact information for a data access committee, ethics committee, or other institutional body to which data requests may be sent. If data are owned by a third party, please indicate how others may request data access.

5. PLOS requires an ORCID iD for the corresponding author in Editorial Manager on papers submitted after December 6th, 2016. Please ensure that you have an ORCID iD and that it is validated in Editorial Manager. To do this, go to ‘Update my Information’ (in the upper left-hand corner of the main menu), and click on the Fetch/Validate link next to the ORCID field. This will take you to the ORCID site and allow you to create a new iD or authenticate a pre-existing iD in Editorial Manager.

6. Your ethics statement should only appear in the Methods section of your manuscript. If your ethics statement is writtenin any section besides the Methods, please delete it from any other section.

7. We notice that your supplementary materials are included in the manuscript file. Please remove them and upload them with the file type 'Supporting Information'. Please ensure that each Supporting Information file has a legend listed in the manuscript after the references list.

8. If the reviewer comments include a recommendation to cite specific previously published works, please review and evaluate these publications to determine whether they are relevant and should be cited. There is no requirement to cite these works unless the editor has indicated otherwise.

**Additional Editor Comments:**

Finally the reviewers have come to a conclusion. It took some extra time to come to an agreement. Thanks for you patience.

[Note: HTML markup is below. Please do not edit.]

Reviewers' comments:

Reviewer's Responses to Questions

**Comments to the Author**

1. Is the manuscript technically sound, and do the data support the conclusions?

The manuscript must describe a technically sound piece of scientific research with data that supports the conclusions. Experiments must have been conducted rigorously, with appropriate controls, replication, and sample sizes. The conclusions must be drawn appropriately based on the data presented.

Reviewer #1: Yes

Reviewer #2: Partly

**********

2. Has the statistical analysis been performed appropriately and rigorously?

Reviewer #1: Yes

Reviewer #2: Yes

**********

3. Have the authors made all data underlying the findings in their manuscript fully available?

The PLOS Data policy requires authors to make all data underlying the findings described in their manuscript fully available without restriction, with rare exception (please refer to the Data Availability Statement in the manuscript PDF file). The data should be provided as part of the manuscript or its supporting information, or deposited to a public repository. For example, in addition to summary statistics, the data points behind means, medians and variance measures should be available. If there are restrictions on publicly sharing data—e.g. participant privacy or use of data from a third party—those must be specified.

Reviewer #1: Yes

Reviewer #2: Yes

**********

4. Is the manuscript presented in an intelligible fashion and written in standard English?

PLOS ONE does not copyedit accepted manuscripts, so the language in submitted articles must be clear, correct, and unambiguous. Any typographical or grammatical errors should be corrected at revision, so please note any specific errors here.

Reviewer #1: Yes

Reviewer #2: Yes

**********

5. Review Comments to the Author

Please use the space provided to explain your answers to the questions above. You may also include additional comments for the author, including concerns about dual publication, research ethics, or publication ethics. (Please upload your review as an attachment if it exceeds 20,000 characters)

Reviewer #1: This is an important study, with a clear message. Especially full HPSF intervention (both healthy lunches and structured PA sessions at school during 4 years) had a strong positive effect on children's (aged 6-12 years) mathematical performance in Netherlands (Parkstad region). This is especially important, because this region is known as a low to moderate socioeconomic area and as the authors state: Equity in education at primary school is an important issue in education, where it is aimed that all students are given equal opportunities to develop their talents, regardless of their SES, ethnicity, or other circumstances. Unfortunately the authors could not analyse the effect of SES-background on the findings, because of acceptable reasons.

The authors hope that the study would be replicated in other SES environments and in other countries, I agree with this.

Reviewer #2: This manuscript by van Engelen et al. describes a nutrition and physical activity intervention in Dutch schools. The primary outcome was an improvement in math and reading scores compared to control schools. They showed that, in schools that had both a free lunch and additional physical activity intervention, students had higher math scores throughout the four-year period and higher reading scores in the first year of intervention.

Overall, this tackles an important question in the field—does providing health-targeted interventions in school improve academic results?

There are some weaknesses here to address. First, it’s unclear how the schools were chosen to be part of the intervention, partial intervention or control groups. Second, the major confounders for academic performance were not adequately measured. For those two reasons, it’s difficult for the reader to decide if the improvements in math and reading scores are related to the intervention or driven by another unmeasured/unaccounted for variable, namely the many socioeconomic drivers of academic performance. Another challenge here is that the reader is not well-guided to understand the degree of improvement in the academic scores. The improvements are statistically significant, but what does a low-to-moderate effect size mean in the real world? How does this compare to other interventions? How does this compare to the effects of SES factors?

It is admirable that a nutrition and movement intervention was studied for its effect on academic performance. Data like this should be more available for educators, school administrators and governments. However, this may be more suited as a quality improvement publication, perhaps in an education-focused journal.

**********

6. PLOS authors have the option to publish the peer review history of their article (what does this mean?). If published, this will include your full peer review and any attached files.

If you choose “no”, your identity will remain anonymous but your review may still be made public.

**Do you want your identity to be public for this peer review?**     For information about this choice, including consent withdrawal, please see our Privacy Policy.

Reviewer #1: No

Reviewer #2: No

**********

[NOTE: If reviewer comments were submitted as an attachment file, they will be attached to this email and accessible via the submission site. Please log into your account, locate the manuscript record, and check for the action link "View Attachments". If this link does not appear, there are no attachment files.]

While revising your submission, please upload your figure files to the Preflight Analysis and Conversion Engine (PACE) digital diagnostic tool, https://pacev2.apexcovantage.com/. PACE helps ensure that figures meet PLOS requirements. To use PACE, you must first register as a user. Registration is free. Then, login and navigate to the UPLOAD tab, where you will find detailed instructions on how to use the tool. If you encounter any issues or have any questions when using PACE, please email PLOS at figures@plos.org. Please note that Supporting Information files do not need this step.

Attachment Submitted filename: editPONE-D-24-56254.pdf

# Author comment: Author response to Decision Letter 1

**DOI:** 10.1371/journal.pone.0334638.r002

22 Sep 2025

Manuscript title: Food for Thought? The Effects of the Healthy Primary School of the Future on Children’s Educational Outcomes

Authors: van Engelen BHW, Hahnraths MTH, Winkens B, Schils t, OCP van Schayck

We thank the reviewers and the academic editor for their constructive comments and careful reading of our manuscript. Below we provide a point-by-point response. Reviewer comments are shown in italics, and our replies follow each point. All suggested changes have been incorporated into the revised manuscript.

Reviewer #1

This is an important study, with a clear message. Especially full HPSF intervention (both healthy lunches and structured PA sessions at school during 4 years) had a strong positive effect on children's (aged 6-12 years) mathematical performance in Netherlands (Parkstad region). This is especially important, because this region is known as a low to moderate socioeconomic area and as the authors state: Equity in education at primary school is an important issue in education, where it is aimed that all students are given equal opportunities to develop their talents, regardless of their SES, ethnicity, or other circumstances. Unfortunately the authors could not analyse the effect of SES-background on the findings, because of acceptable reasons.

The authors hope that the study would be replicated in other SES environments and in other countries, I agree with this.

Response:

We thank the reviewer for the positive and supportive feedback. We agree that equity in education is a key issue and that replication in other socioeconomic contexts is needed. As noted, we could not include SES in the analyses. This was not due to poor documentation, but because SES information in Dutch primary schools is only available via parental questionnaires, which had a ~50% non-response rate. Non-response is known to be common among families with lower SES or non-Dutch language backgrounds (Boudewijns et al., 2019). This resulted in too many missing values to allow for reliable adjustment or multiple imputation. While multiple imputations are technically possible even with a high percentage of missing data (e.g., 50%), its reliability depends on having other variables that are strongly correlated with the variable containing the missing values. In this case, the outcome variable is likely related to socioeconomic status (SES), but not strongly enough to produce reliable imputations. We also emphasize that all schools were located in the same low-to-moderate SES region under one school board, which reduces but does not eliminate SES heterogeneity. We have clarified this in the revised Strengths and Limitations section by adding a paragraph (page 12, lines 330–341):

‘The reason why we were unable to adjust for children’s socioeconomic background was that information on parental education (as a proxy for SES) is not routinely collected in Dutch primary education and was therefore only available via parental questionnaires. Unfortunately, a substantial proportion of parents (~50%) did not complete these questionnaires. Non-response is known to be more common among families with a lower SES or a non-Dutch language background [30]. While residual confounding cannot be excluded, several considerations may reduce this concern: (1) all schools were located in the same low-to-moderate SES region under one school board, limiting SES heterogeneity; and (2) a previous study (specifically designed to investigate the external validity of the study with the present study population [30]) confirmed that participants were broadly representative of the region in terms of sex (48% boys vs. 50% regionally) and socioeconomic status, as indicated by maternal educational level (25% low, 45% middle, 30% high vs. 27%, 42%, 31% regionally), as well as health-related characteristics. Nevertheless, replication of this study in other socioeconomic contexts is necessary to establish the generalizability of our findings.’

We also emphasize that all schools were located in the same low-to-moderate SES region under one school board, which reduces but may not fully eliminate SES heterogeneity.

Reviewer #2

This manuscript by van Engelen et al. describes a nutrition and physical activity intervention in Dutch schools. The primary outcome was an improvement in math and reading scores compared to control schools. They showed that, in schools that had both a free lunch and additional physical activity intervention, students had higher math scores throughout the four-year period and higher reading scores in the first year of intervention.

Overall, this tackles an important question in the field—does providing health-targeted interventions in school improve academic results?

There are some weaknesses here to address. First, it’s unclear how the schools were chosen to be part of the intervention, partial intervention or control groups. Second, the major confounders for academic performance were not adequately measured. For those two reasons, it’s difficult for the reader to decide if the improvements in math and reading scores are related to the intervention or driven by another unmeasured/unaccounted for variable, namely the many socioeconomic drivers of academic performance. Another challenge here is that the reader is not well-guided to understand the degree of improvement in the academic scores. The improvements are statistically significant, but what does a low-to-moderate effect size mean in the real world? How does this compare to other interventions? How does this compare to the effects of SES factors?

It is admirable that a nutrition and movement intervention was studied for its effect on academic performance. Data like this should be more available for educators, school administrators and governments. However, this may be more suited as a quality improvement publication, perhaps in an education-focused journal.

Response:

We thank the reviewer for the thoughtful review and for recognizing the importance of our research question. We appreciate your positive feedback on our manuscript, which investigates the impact of a nutrition and physical activity intervention on academic performance in Dutch schools.

We are glad you found our study's aim of examining whether health-targeted interventions in schools can improve academic results to be an important contribution to the field. Your summary of our key findings—that schools with both a free lunch and additional physical activity intervention saw sustained improvements in math scores and initial improvements in reading scores—accurately reflects the core results of our work.

Your comments are highly valuable and will help us improve the final manuscript. We are grateful for your time and expertise in reviewing our work. Please find below our response to your concerns.

1. It’s unclear how the schools were chosen to be part of the intervention, partial intervention or control groups.

Response:

We thank the reviewer for this comment. We would like to clarify that our study employed a longitudinal quasi-experimental design rather than a randomized controlled trial (RCT). We have now explicitly stated this in the Abstract (line 26), Methods (line 123), and Discussion (line 318). An RCT was not feasible in this context. Randomization on individual level was not possible as it would have required randomizing children across different schools. In addition, successful implementation depended on the cooperation and commitment of school boards, principals, teachers, and parents. For these reasons, voluntary participation at the school level was the only realistic option, making a quasi-experimental design the most appropriate and feasible choice. This is explained in detail in our publication describing the protocol and study design of this study [24]. We have also added this explanation in the Discussion line 381-384 to justify our methodological approach.

‘While some may view school health programs primarily as quality improvement, we emphasize that the HPSF was evaluated in a longitudinal quasi-experimental design with repeated, standardized national testing over four years. This methodological rigor, combined with a large dataset and robust analyses, allows us to provide scientific evidence on the academic effects of a complex school-based health intervention.’

2. The major confounders for academic performance were not adequately measured.

Response:

We agree that SES and other potential confounders are important. We actively sought SES data, but in Dutch primary education this information is only available through parental questionnaires. Unfortunately, about 50% of parents did not respond, and non-response was more common among families with a lower SES or a non-Dutch language background (Boudewijns et al., 2019: as explained, this previous study specifically designed to investigate the external validity of the study with the present study population [30]). This limitation reflects parental non-response and resulted in too many missing values for meaningful inclusion in our analyses.

Nevertheless, several considerations reduce the risk of bias. First, all schools were located in the same low-to-moderate SES region under one school board, which limits heterogeneity between schools. Second, Boudewijns et al. (2019) showed that participants in the HPSF trial were broadly representative of the regional school population: 25% of mothers had a low education level, 45% a middle level, and 30% a high level, compared with 27%, 42%, and 31% regionally. In terms of sex distribution, the sample (48% boys) was also comparable to the regional population (50% boys). Finally, health-related characteristics of participating children were consistent with regional averages.

Taken together, these findings suggest that although individual-level SES data were incomplete, the study population is likely representative of the wider regional context, thereby reducing the risk of systematic bias. In line with Reviewer 1’s comment, we note that the inability to analyze SES effects stems from well-documented reasons. Reviewer 1 also acknowledged this point and agreed that replication of the study in other SES environments and in other countries is important, and we explicitly highlight this recommendation in the revised manuscript. We have clarified and expanded this limitation in the revised manuscript by adding a paragraph to the discussion section (page 12, lines 330–341):

‘The reason why we were unable to adjust for children’s socioeconomic background was that information on parental education (as a proxy for SES) is not routinely collected in Dutch primary education and was therefore only available via parental questionnaires. Unfortunately, a substantial proportion of parents (~50%) did not complete these questionnaires. Non-response was more common among families with a lower SES or a non-Dutch language background [30]. This resulted in too many missing values to allow for meaningful inclusion of SES in the analyses. While residual confounding cannot be excluded, several considerations may reduce this concern: (1) all schools were located in the same low-to-moderate SES region under one school board, limiting SES heterogeneity; and (2) a previous study (specifically designed to investigate the external validity of the study with the present study population [30]) confirmed that participants were broadly representative of the region in terms of sex (48% boys vs. 50% regionally) and socioeconomic status, as indicated by maternal educational level (25% low, 45% middle, 30% high vs. 27%, 42%, 31% regionally), as well as health-related characteristics. Nevertheless, replication of this study in other socioeconomic contexts is necessary to establish the generalizability of our findings.’

3. The reader is not well-guided to understand the degree of improvement in the academic scores. The improvements are statistically significant, but what does a low-to-moderate effect size mean in the real world? How does this compare to other interventions? How does this compare to the effects of SES factors?

Response:

We appreciate this important comment. To facilitate interpretation, we presented all results in standardized effect sizes (Cohen’s d). In the revised manuscript, we have expanded the interpretation by providing a theoretical example:

• A child in a control school gained on average 12.9 mathematics points per year, compared to 16.5 points in a full HPSF school.

• The national average was 15.5 points per year.

• Thus, children in HPSF schools progressed faster than the national trend, while those in control schools fell behind.

To further illustrate this, we have added a new figure to Appendix III, showing the mathematics performance change after one year in control schools, full HPSF schools, and the national average. Although the standardized effect sizes were small-to-moderate in statistical terms, such differences may accumulate meaningful educational consequences. Direct comparison with other health-based interventions is unfortunately not possible, as no comparable studies have been published with standardized test outcomes. Likewise, we cannot directly compare SES effects in this dataset due to missing data. These clarifications have been added to the revised Discussion on page 13 line 350-359:

‘Interpretation of effect sizes in educational outcomes was challenging. We therefore presented all results also in standardized form (Cohen’s d) to facilitate comparison. To further contextualize the findings, we illustrated the effects using a theoretical example based on observed data: Children in control schools gained on average 12.9 mathematics points per year, compared to 16.5 points in full HPSF schools per year, while the national average was 15.5 points per year. Thus, children in HPSF schools progressed faster than the national trend, whereas those in control schools fell behind (Appendix III). Although the standardized effect sizes were small-to-moderate in statistical terms, such differences may accumulate over years to have meaningful educational consequences. Direct comparison with other health-based interventions is unfortunately not possible, as to our knowledge no comparable studies with standardized test outcomes have been published.

4. This may be more suited as a quality improvement publication, perhaps in an education-focused journal.

Response:

We respectfully disagree. The Healthy Primary School of the Future intervention was evaluated using a longitudinal quasi-experimental design with repeated national standardized tests over four years, involving rigorous statistical analysis. This goes beyond educational quality improvement reporting and provides scientific evidence on the academic impact of a complex school-based health intervention. Importantly, a previous study reporting on the long-term health effects of HPSF by Willeboordse et al. (2022) was earlier published in PLOS ONE, demonstrating that this journal considers school-based interventions with rigorous quasi-experimental designs suitable for its scope. According to the PLOS ONE mission statement, the journal welcomes “primary research from any discipline within science and medicine” that is methodologically rigorous and ethically conducted, regardless of perceived impact. We believe that our study clearly meets these criteria, as it applies to a robust design to address a relevant question at the intersection of public health, education, and social equity.

Furthermore, to facilitate a more generic interpretation of our outcomes, we have added Appendix III, which illustrates the practical meaning of effect sizes in terms of annual mathematics score gains compared to national averages. We have also strengthened the Implications section of the manuscript to emphasize the broader significance of these findings by adding the following to page 14 line 379-382:

‘While some may view school health programs primarily as quality improvement, we emphasize that the HPSF was evaluated in a longitudinal quasi-experimental design with repeated, standardized national testing over four years. This methodological rigor, combined with a large dataset and robust analyses, allows us to provide scientific evidence on the academic effects of a complex scho

Attachment Submitted filename: rebuttal letter.docx

# Peer review: Decision Letter 1

**DOI:** 10.1371/journal.pone.0334638.r003

**Authors:** Emma Campbell

18 Dec 2025

PONE-D-24-56254R1

Food for thought? The effects of the Healthy Primary School of the Future on children’s educational outcomes

PLOS One

Dear Dr. van Engelen,

Thank you for submitting your manuscript to PLOS ONE. After careful consideration, we feel that it has merit but does not fully meet PLOS ONE’s publication criteria as it currently stands. Therefore, we invite you to submit a revised version of the manuscript that addresses the points raised during the review process.

Thank you for your patience whilst we have tried to address some of the concerns we have previously discussed. Reviewer 3 and 4 have provided some constructive feedback to be incorporated into an additional revision. We appreciate that this is frustrating but do feel that due to the wide implications and potential impact of this study it was necessary to secure some additional feedback. Once these comments have been addressed we hope the study will be ready to proceed back towards publication.

Please ensure that you respond to the comments from Reviewers 3 and 4 and make any revisions necessary to address their concerns, we specifically want you to focus on any comments around the statistical analysis from Reviewer 4.

Please submit your revised manuscript by Jan 30 2026 11:59PM. If you will need more time than this to complete your revisions, please reply to this message or contact the journal office at plosone@plos.org. When you're ready to submit your revision, log on to https://www.editorialmanager.com/pone/ and select the 'Submissions Needing Revision' folder to locate your manuscript file.

Please include the following items when submitting your revised manuscript:

- A letter that responds to each point raised by the academic editor and reviewer(s). You should upload this letter as a separate file labeled 'Response to Reviewers'.
- A marked-up copy of your manuscript that highlights changes made to the original version. You should upload this as a separate file labeled 'Revised Manuscript with Track Changes'.
- An unmarked version of your revised paper without tracked changes. You should upload this as a separate file labeled 'Manuscript'.

If you would like to make changes to your financial disclosure, please include your updated statement in your cover letter. Guidelines for resubmitting your figure files are available below the reviewer comments at the end of this letter.

If applicable, we recommend that you deposit your laboratory protocols in protocols.io to enhance the reproducibility of your results. Protocols.io assigns your protocol its own identifier (DOI) so that it can be cited independently in the future. For instructions see: https://journals.plos.org/plosone/s/submission-guidelines#loc-laboratory-protocols. Additionally, PLOS ONE offers an option for publishing peer-reviewed Lab Protocol articles, which describe protocols hosted on protocols.io. Read more information on sharing protocols at https://plos.org/protocols?utm_medium=editorial-email&utm_source=authorletters&utm_campaign=protocols.

We look forward to receiving your revised manuscript.

Kind regards,

Emma Campbell, Ph.D

Staff Editor

PLOS One

Journal Requirements:

If the reviewer comments include a recommendation to cite specific previously published works, please review and evaluate these publications to determine whether they are relevant and should be cited. There is no requirement to cite these works unless the editor has indicated otherwise.

[Note: HTML markup is below. Please do not edit.]

Reviewers' comments:

Reviewer's Responses to Questions

**Comments to the Author**

1. If the authors have adequately addressed your comments raised in a previous round of review and you feel that this manuscript is now acceptable for publication, you may indicate that here to bypass the “Comments to the Author” section, enter your conflict of interest statement in the “Confidential to Editor” section, and submit your "Accept" recommendation.

Reviewer #1: All comments have been addressed

Reviewer #2: All comments have been addressed

Reviewer #3: All comments have been addressed

Reviewer #4: (No Response)

**********

2. Is the manuscript technically sound, and do the data support the conclusions?

The manuscript must describe a technically sound piece of scientific research with data that supports the conclusions. Experiments must have been conducted rigorously, with appropriate controls, replication, and sample sizes. The conclusions must be drawn appropriately based on the data presented.

Reviewer #1: Yes

Reviewer #2: Yes

Reviewer #3: Yes

Reviewer #4: Partly

**********

3. Has the statistical analysis been performed appropriately and rigorously?

Reviewer #1: Yes

Reviewer #2: Yes

Reviewer #3: Yes

Reviewer #4: No

**********

4. Have the authors made all data underlying the findings in their manuscript fully available?

The PLOS Data policy requires authors to make all data underlying the findings described in their manuscript fully available without restriction, with rare exception (please refer to the Data Availability Statement in the manuscript PDF file). The data should be provided as part of the manuscript or its supporting information, or deposited to a public repository. For example, in addition to summary statistics, the data points behind means, medians and variance measures should be available. If there are restrictions on publicly sharing data—e.g. participant privacy or use of data from a third party—those must be specified.

Reviewer #1: Yes

Reviewer #2: Yes

Reviewer #3: Yes

Reviewer #4: Yes

**********

5. Is the manuscript presented in an intelligible fashion and written in standard English?

PLOS ONE does not copyedit accepted manuscripts, so the language in submitted articles must be clear, correct, and unambiguous. Any typographical or grammatical errors should be corrected at revision, so please note any specific errors here.

Reviewer #1: Yes

Reviewer #2: Yes

Reviewer #3: Yes

Reviewer #4: Yes

**********

6. Review Comments to the Author

Please use the space provided to explain your answers to the questions above. You may also include additional comments for the author, including concerns about dual publication, research ethics, or publication ethics. (Please upload your review as an attachment if it exceeds 20,000 characters)

Reviewer #1: All comments given by me and the other reviewer have been treated satisfactorily. In fact i was ready to accept the paper already at earlier phase.

Reviewer #2: The authors have done an admirable job of responding to the comments of both the first reviewer and myself. I find their answers to our questions thoughtful and the additions to the manuscript are all well-done. The lack of SES data is now much more clear, and the additional text is helpful. I found the discussion on how test scores compare to national averages very helpful for context.

Reviewer #3: Dear Authors,

The manuscript presents a valuable study examining the effects of a school-based health intervention on academic performance using longitudinal data. The large sample size, the use of national standardized tests, and the application of marginal models constitute important strengths of the work. The findings regarding mathematics performance are particularly relevant for policymakers.

However, several methodological aspects require clarification:

Study design: The intervention was not implemented by the researchers, and schools voluntarily opted into the full HPSF, partial HPSF, or control condition. This places the study closer to a natural experiment / observational longitudinal comparative design than to a classical quasi-experimental framework. This methodological distinction should be articulated more clearly in the discussion.

Confounding variables: Key covariates such as SES and the home learning environment could not be included due to substantial missing data. This raises the possibility of selection bias and limits causal inferences. A stronger acknowledgment of this limitation is recommended.

Reading comprehension outcomes: The results are inconsistent across years, and the absence of baseline data weakens interpretability. An expanded discussion of why partial HPSF schools outperform in certain years would be beneficial.

Intervention fidelity: Information on the consistency of implementing the healthy lunch and physical activity components over the four-year period would enhance the interpretability of the findings.

Overall, the study makes a meaningful contribution to the field. With minor revisions addressing the points above, the manuscript would be suitable for publication.

Recommendation: Minor Revision

Sincerely.

Reviewer #4: The manuscript requires further improvement.

It is recommended that the manuscript undergo English proofreading by a native speaker to improve clarity and readability.

Line 123, Line 145-146: The sentence requires revision.

Line 188-189: The sentence could be improved e.g. Proportions were compared using Pearson’s chi-square tests, and mean values were compared using one-way ANOVA.

Line 189-190: The sentence requires revision e.g. If the homoscedasticity assumption (assessed using Levene’s test) was violated, Welch’s test was used for overall comparisons, and the Games-Howell test was used for pairwise comparisons.

Line 193 -197: The statistical test is to be stated. If GEE is employed, the purpose is to clearly highlight, e.g, to estimate the average intervention effect across all participants.

Line 202-204: The sentence requires revision. e.g. 'A marginal model for repeated measures was used, as it included all available data and assumed that missing values were missing at random (MAR). Estimation was performed using a restricted maximum likelihood (REML) approach.' The exact name of the statistical test is to be mentioned e.g. LMM, MMRM etc. The sentence is to tailor the specific statistical test that was employed.

Line 204-205: For the corrected mean difference, state it is adjusted for variables such as…

Line 209: The sentence could be improved. e.g. To adjust for multiple group comparisons, the Bonferroni method was applied.

Line 214, 217 etc: ‘was made’ to be rephrased as ‘was conducted’.

Line 227: The time point to be stated. Alignment of the figures requires cosmetic changes.

Line 255, Line 262, Line 547: The Δ1–Δ4 may be confusing, Suggest to highlight it as Δ1, Δ2, Δ3, Δ4

In the statistical analyses section of the Methods, ensure that all statistical tests mentioned and utilised are clearly indicated in the Results (e.g., in table or figure footnotes). Where applicable, include a statement confirming that the assumptions of these tests were assessed and fulfilled.

For any statistical tests or analyses described in the Methods, any important details or main output from the statistical software that are not presented in the main manuscript are to be provided as supplementary files.

A flow chart is to be included, if possible, to illustrate how participants were selected and allocated to the intervention groups in this manuscript.

The numbering for each title/sub title is to be omitted.

**********

7. PLOS authors have the option to publish the peer review history of their article (what does this mean?). If published, this will include your full peer review and any attached files.

If you choose “no”, your identity will remain anonymous but your review may still be made public.

**Do you want your identity to be public for this peer review?**     For information about this choice, including consent withdrawal, please see our Privacy Policy.

Reviewer #1: **Yes:**    Jorma Viikari

Reviewer #2: No

Reviewer #3: No

Reviewer #4: No

**********

[NOTE: If reviewer comments were submitted as an attachment file, they will be attached to this email and accessible via the submission site. Please log into your account, locate the manuscript record, and check for the action link "View Attachments". If this link does not appear, there are no attachment files.]

To ensure your figures meet our technical requirements, please review our figure guidelines: https://journals.plos.org/plosone/s/figures

You may also use PLOS’s free figure tool, NAAS, to help you prepare publication quality figures: https://journals.plos.org/plosone/s/figures#loc-tools-for-figure-preparation.

NAAS will assess whether your figures meet our technical requirements by comparing each figure against our figure specifications.

# Author comment: Author response to Decision Letter 2

**DOI:** 10.1371/journal.pone.0334638.r004

23 Feb 2026

Response to Reviewers 3 and 4

We thank Reviewers 3 and 4 for their careful reading of the manuscript and their constructive feedback. We also thank the editor for the opportunity to revise the manuscript. Below, we address each comment point-by-point. Revisions have been made in close consultation with the statistical expert on the author team (Professor Bjorn Winkens) and are reflected in the revised manuscript.

1. Study design clarification

Reviewer comment:

The study more closely resembles a natural experiment / observational longitudinal design rather than a classical quasi-experimental study.

Response:

We thank the reviewer for this important comment and appreciate the opportunity to further clarify the study design. While schools indeed participated on a voluntary basis and allocation was not randomized, the study was intentionally designed and implemented as a quasi-experimental study, consistent with the original HPSF study protocol (Willeboordse et al., 2016). The intervention conditions (full HPSF, partial HPSF, control), timing of implementation, predefined outcomes, and longitudinal follow-up were specified a priori and systematically evaluated, which aligns with established definitions of quasi-experimental designs. Moreover, the full result paper presenting all obesity-related outcomes of the intervention in this quasi-experimental study was earlier published in PlosOne (Willeboordse et al, 2022) and was presented as such. No other reviewers in this as well as the preceding peer-review by PlosOne have ever suggested to relabel our study as a natural experiment or observational study.

At the same time, we agree that the absence of random allocation limits causal inference. To address this, we have clarified in the Discussion that, although the study follows a quasi-experimental framework, results should be interpreted with appropriate caution regarding causality.

Suggested manuscript text (Limitations page 13 lines 337-345)

Implementation of the core components of the HPSF, including the healthy lunch and structured physical activity, was monitored through direct and intensive involvement of the research team, with regular contact at both the school and school board levels to assess whether implementation aligned with the agreed implementation plan. This multi-level approach was intended to address a well-known challenge in educational research, namely that interventions are frequently not implemented as intended. Engagement at both the school and governance levels supported internal ownership of the intervention and facilitated sustained implementation over time. Moreover, consistent with the conceptualization of the HPSF as a complex adaptive system, the accompanying process evaluation emphasized not only fidelity but also context-specific adaptations, as strict fidelity does not necessarily translate into greater effectiveness in complex educational settings

2. Confounding variables and selection bias

Reviewer comment:

The absence of key covariates such as SES and the home learning environment raises concerns about selection bias and limits causal inference.

Response:

We agree with the reviewer. Unfortunately, the substantial amount of missing data on SES and home learning environment variables precluded their inclusion in the final models. While this may indeed introduce residual confounding and selection bias, we included all relevant variables that could be reliably analyzed within the scope of the present study.

Revision made:

We have strengthened the limitations section by explicitly discussing the potential impact of unmeasured confounding and selection bias, and by tempering causal language throughout the manuscript.

Suggested manuscript text (Limitations page 14 lines 375-383)

“An important limitation is that key potential confounders, such as socioeconomic status, the home learning environment and comprehension reading at baseline, could not be included due to substantial missing data. As a result, residual confounding and selection bias cannot be ruled out. Schools opting into the HPSF may differ systematically from control schools in unobserved characteristics related to both health promotion and academic outcomes, which limits causal interpretation of the findings.

Previous research on the HPSF/GBT cohort indicates that although evidence for a healthy-volunteer effect is limited, responders were more likely to have higher educated parents and differed from the national population on several sociodemographic characteristics. Consequently, external validity appears to be high with respect to the regional population but more limited when comparisons are made at the national level.(30)’’

3. Reading comprehension outcomes

Reviewer comment:

Results for reading comprehension are inconsistent across years, and the lack of baseline data complicates interpretation. Further discussion of why partial HPSF schools outperform in some years is recommended.

Response:

The inconsistency in the results across years indeed complicates the interpretation. We have therefore expanded the Discussion to more explicitly address this variability and to propose plausible explanations for the relatively better performance of partial HPSF schools in certain years.

Suggested manuscript text (Limitations page 12 rules 315-319):

As for the larger effect of partial HPSF over full HPSF, this difference was only statistically significant at year 4 and showed no consistent pattern (almost no difference at year 1 and year 3). This difference over time might also be partially explained by a possible difference between the partial and full HPSF schools at baseline, which could not be accounted for due to the number of missing values. This is also mentioned as one of the main limitations below.

4. Intervention fidelity

Reviewer comment:

More information on the consistency of implementing the healthy lunch and physical activity components over the four-year period would improve interpretability.

Response:

We thank the reviewer for this important observation. Indeed, many educational interventions are not implemented as originally intended, which can substantially affect interpretability of findings. In the present study, implementation of the core components (healthy lunch and structured physical activity) was therefore closely monitored through direct and intensive involvement of the research team, including regular contact with schools to assess adherence to the implementation plan. In addition, monitoring occurred not only at the school level but also via close collaboration with school boards, thereby embedding the intervention within existing governance structures and enhancing internal ownership. This multi-level engagement has now been more clearly described in the Discussion section.

Suggested manuscript text (Limitations page 13 lines 337-345)

Implementation of the core components of the HPSF, including the healthy lunch and structured physical activity, was monitored through direct and intensive involvement of the research team, with regular contact at both the school and school board levels to assess whether implementation aligned with the agreed implementation plan. This multi-level approach was intended to address a well-known challenge in educational research, namely that interventions are frequently not implemented as intended. Engagement at both the school and governance levels supported internal ownership of the intervention and facilitated sustained implementation over time. Moreover, consistent with the conceptualization of the HPSF as a complex adaptive system, the accompanying process evaluation emphasized not only fidelity but also context-specific adaptations, as strict fidelity does not necessarily translate into greater effectiveness in complex educational settings

Reviewer #4

We thank Reviewer #4 for the detailed and technically oriented comments, which substantially improved the clarity and rigor of the manuscript.

1. Line-by-line statistical clarifications (Lines 123–217)

Comment (Lines 123, 145–146):

The sentence requires revision.

Response:

The indicated sentences have been revised to improve clarity and readability. If further revisions are needed, we are more than willing to accommodate this.

Comment (Lines 188–189):

The sentence could be improved, e.g. Proportions were compared using Pearson’s chi-square tests, and mean values were compared using one-way ANOVA.

Response:

The text has been revised accordingly, and the statistical tests are now explicitly stated.

Comment (Lines 189–190):

The sentence requires revision, e.g. If the homoscedasticity assumption (assessed using Levene’s test) was violated, Welch’s test was used for overall comparisons, and the Games–Howell test was used for pairwise comparisons.

Response:

The text has been revised accordingly to clearly describe the assessment of homoscedasticity and the use of Welch’s and Games–Howell tests when this assumption was violated.

Comment (Lines 193–197):

The statistical test is to be stated. If GEE is employed, the purpose should be clearly highlighted (e.g., to estimate the average intervention effect across all participants).

Response:

The statistical approach has been clarified. The exact model used is now explicitly stated, and its purpose—estimating average intervention effects over time—has been clearly described.

Comment (Lines 202–204):

The sentence requires revision. The exact name of the statistical test (e.g. LMM, MMRM) should be mentioned, and the sentence should be tailored to the specific test employed.

Response:

This section has been revised accordingly. The analysis is now explicitly described as a mixed model for repeated measures (MMRM), and the description of the model and estimation approach has been clarified.

Comment (Lines 204–205):

For the corrected mean difference, state that it is adjusted for variables such as…

Response:

The manuscript now explicitly states which covariates were included in the adjustment of corrected mean differences (i.e. age, sex, test version, and baseline score for mathematics).

Comment (Line 209):

The sentence could be improved, e.g. To adjust for multiple group comparisons, the Bonferroni method was applied.

Response:

The sentence has been revised to:

“To adjust for multiple group comparisons, the Bonferroni method was applied (significance level α = 0.05/3 = 0.0167).”

Comment (Lines 214, 217, etc.):

“Was made” should be rephrased as “was conducted”.

Response:

This has been revised throughout the manuscript.

Comment (Line 227):

The time point should be stated. Alignment of the figures requires cosmetic changes.

Response:

We thank the reviewer for this comment. However, it was not entirely clear to us which specific time point or figure alignment issue this comment refers to. We have therefore carefully checked the manuscript and figures and confirmed that all time points are clearly indicated and that the figures are consistently aligned. We would be happy to address this further should the reviewer be able to clarify the comment.

Comment (Lines 255, 262, 547):

The Δ1–Δ4 notation may be confusing. It is suggested to highlight this as Δ1, Δ2, Δ3, Δ4.

Response:

This notation has been clarified throughout the text, tables, and figure captions.

Comment:

In the statistical analyses section of the Methods, ensure that all statistical tests mentioned are clearly indicated in the Results, and include a statement confirming that assumptions were assessed and fulfilled.

Response:

Consistency between the Methods and Results sections has been ensured. All statistical tests described in the Methods are explicitly reported in the Results (e.g. Pearson’s chi-square test, Welch’s test, Games–Howell test). In addition, the following statements were added:

• Methods (blz en regels toevoegen):

“The normality assumption of the residuals was checked per group and per timepoint (exposure) using histograms and Q–Q plots.”

• Results (blz en regels toevoegen:

“All intervention effects were assessed using mixed models for repeated measures (MMRM), and all model assumptions were met.”

Comment:

For any statistical tests or analyses described in the Methods, any important details or main output not presented in the main manuscript should be provided as supplementary files.

Response:

Important details were added to the manuscript (Pearson chi-square value, Welch F-value) as well as to a supplementary table (Mixed model for repeated measures results, including the overall F-test values and p-values for the interaction effect between group and exposure as well as the variance-covariance matrix of the repeated measures).

Comment:

A flow chart should be included, if possible, to illustrate how participants were selected and allocated to the intervention groups.

Response:

A flow chart illustrating participant selection and allocation to intervention groups has been added to the Supplementary Materials.

Comment:

The numbering for each title/subtitle should be omitted.

Response:

All numbering of titles and subtitles has been removed in accordance with journal formatting requirements.

Closing statement

We believe these revisions substantially improve methodological transparency, statistical clarity, and interpretability of the findings. We thank Reviewers 3 and 4 for their constructive feedback, which has strengthened the manuscript.

Attachment Submitted filename: Response to Reviewers 3 and 4.docx

# Peer review: Decision Letter 2

**DOI:** 10.1371/journal.pone.0334638.r005

**Authors:** Marianne Clemence

12 Mar 2026

Food for thought? The effects of the Healthy Primary School of the Future on children’s educational outcomes

PONE-D-24-56254R2

Dear Dr. van Engelen,

We’re pleased to inform you that your manuscript has been judged scientifically suitable for publication and will be formally accepted for publication once it meets all outstanding technical requirements.

Within one week, you’ll receive an e-mail detailing the required amendments. When these have been addressed, you’ll receive a formal acceptance letter and your manuscript will be scheduled for publication.

An invoice will be generated when your article is formally accepted. Please note, if your institution has a publishing partnership with PLOS and your article meets the relevant criteria, all or part of your publication costs will be covered. Please make sure your user information is up-to-date by logging into Editorial Manager at Editorial Manager® and clicking the ‘Update My Information' link at the top of the page. For questions related to billing, please contact billing support.

If your institution or institutions have a press office, please notify them about your upcoming paper to help maximize its impact. If they’ll be preparing press materials, please inform our press team as soon as possible -- no later than 48 hours after receiving the formal acceptance. Your manuscript will remain under strict press embargo until 2 pm Eastern Time on the date of publication. For more information, please contact onepress@plos.org.

Kind regards,

Marianne Clemence

Staff Editor

PLOS One

Additional Editor Comments (optional):

Reviewers' comments:

Reviewer's Responses to Questions

**Comments to the Author**

1. If the authors have adequately addressed your comments raised in a previous round of review and you feel that this manuscript is now acceptable for publication, you may indicate that here to bypass the “Comments to the Author” section, enter your conflict of interest statement in the “Confidential to Editor” section, and submit your "Accept" recommendation.

Reviewer #3: All comments have been addressed

Reviewer #4: All comments have been addressed

**********

2. Is the manuscript technically sound, and do the data support the conclusions?

The manuscript must describe a technically sound piece of scientific research with data that supports the conclusions. Experiments must have been conducted rigorously, with appropriate controls, replication, and sample sizes. The conclusions must be drawn appropriately based on the data presented.

Reviewer #3: Yes

Reviewer #4: (No Response)

**********

3. Has the statistical analysis been performed appropriately and rigorously?

Reviewer #3: Yes

Reviewer #4: (No Response)

**********

4. Have the authors made all data underlying the findings in their manuscript fully available?

The PLOS Data policy requires authors to make all data underlying the findings described in their manuscript fully available without restriction, with rare exception (please refer to the Data Availability Statement in the manuscript PDF file). The data should be provided as part of the manuscript or its supporting information, or deposited to a public repository. For example, in addition to summary statistics, the data points behind means, medians and variance measures should be available. If there are restrictions on publicly sharing data—e.g. participant privacy or use of data from a third party—those must be specified.

Reviewer #3: Yes

Reviewer #4: (No Response)

**********

5. Is the manuscript presented in an intelligible fashion and written in standard English?

PLOS ONE does not copyedit accepted manuscripts, so the language in submitted articles must be clear, correct, and unambiguous. Any typographical or grammatical errors should be corrected at revision, so please note any specific errors here.

Reviewer #3: Yes

Reviewer #4: (No Response)

**********

6. Review Comments to the Author

Please use the space provided to explain your answers to the questions above. You may also include additional comments for the author, including concerns about dual publication, research ethics, or publication ethics. (Please upload your review as an attachment if it exceeds 20,000 characters)

Reviewer #3: The authors have completed revisions or written compelling explanations. Upon reviewing the revision manuscript, the article is considered acceptable for publication.

Reviewer #4: (No Response)

**********

7. PLOS authors have the option to publish the peer review history of their article (what does this mean?). If published, this will include your full peer review and any attached files.

If you choose “no”, your identity will remain anonymous but your review may still be made public.

**Do you want your identity to be public for this peer review?**     For information about this choice, including consent withdrawal, please see our Privacy Policy.

Reviewer #3: **Yes:**    SEHER ÇETİNKAYA

Reviewer #4: No

**********

# Editor report: Acceptance letter

**DOI:** 10.1371/journal.pone.0334638.r006

**Authors:** Marianne Clemence

PONE-D-24-56254R1

PLOS ONE

Dear Dr. van Engelen,

I'm pleased to inform you that your manuscript has been deemed suitable for publication in PLOS ONE. Congratulations! Your manuscript is now being handed over to our production team.

At this stage, our production department will prepare your paper for publication. This includes ensuring the following:

* All references, tables, and figures are properly cited

* All relevant supporting information is included in the manuscript submission,

* There are no issues that prevent the paper from being properly typeset

You will receive further instructions from the production team, including instructions on how to review your proof when it is ready. Please keep in mind that we are working through a large volume of accepted articles, so please give us a few days to review your paper and let you know the next and final steps.

Lastly, if your institution or institutions have a press office, please let them know about your upcoming paper now to help maximize its impact. If they'll be preparing press materials, please inform our press team within the next 48 hours. Your manuscript will remain under strict press embargo until 2 pm Eastern Time on the date of publication. For more information, please contact onepress@plos.org.

You will receive an invoice from PLOS for your publication fee after your manuscript has reached the completed accept phase. If you receive an email requesting payment before acceptance or for any other service, this may be a phishing scheme. Learn how to identify phishing emails and protect your accounts at https://explore.plos.org/phishing.

If we can help with anything else, please email us at customercare@plos.org.

Thank you for submitting your work to PLOS ONE and supporting open access.

Kind regards,

PLOS ONE Editorial Office Staff

on behalf of

Dr. Stijn Michielse

Academic Editor

PLOS ONE
